# Supplementary figures and images for: The molecular logic of Gtr1/2- and Pib2-dependent TORC1 regulation in budding yeast
Source: eLife. 2025 Jul 7;13:RP94628. doi: 10.7554/eLife.94628 (PMC12234008; doi:10.7554/eLife.94628)

Figure 1A

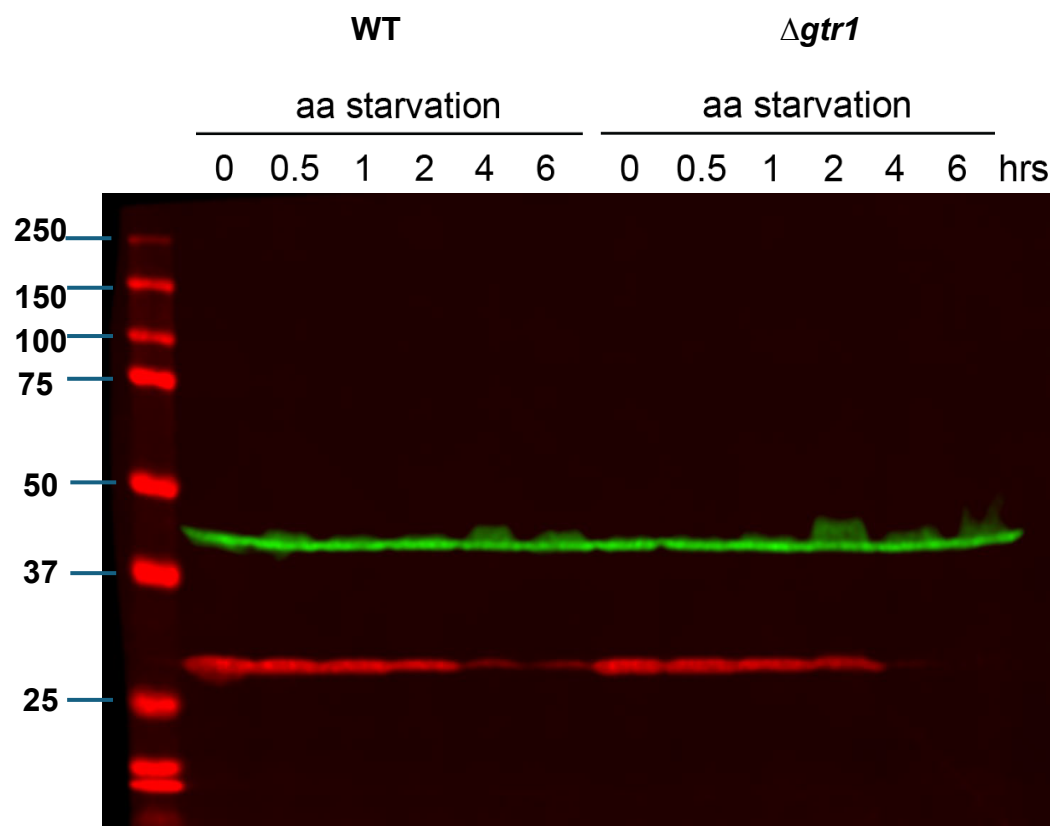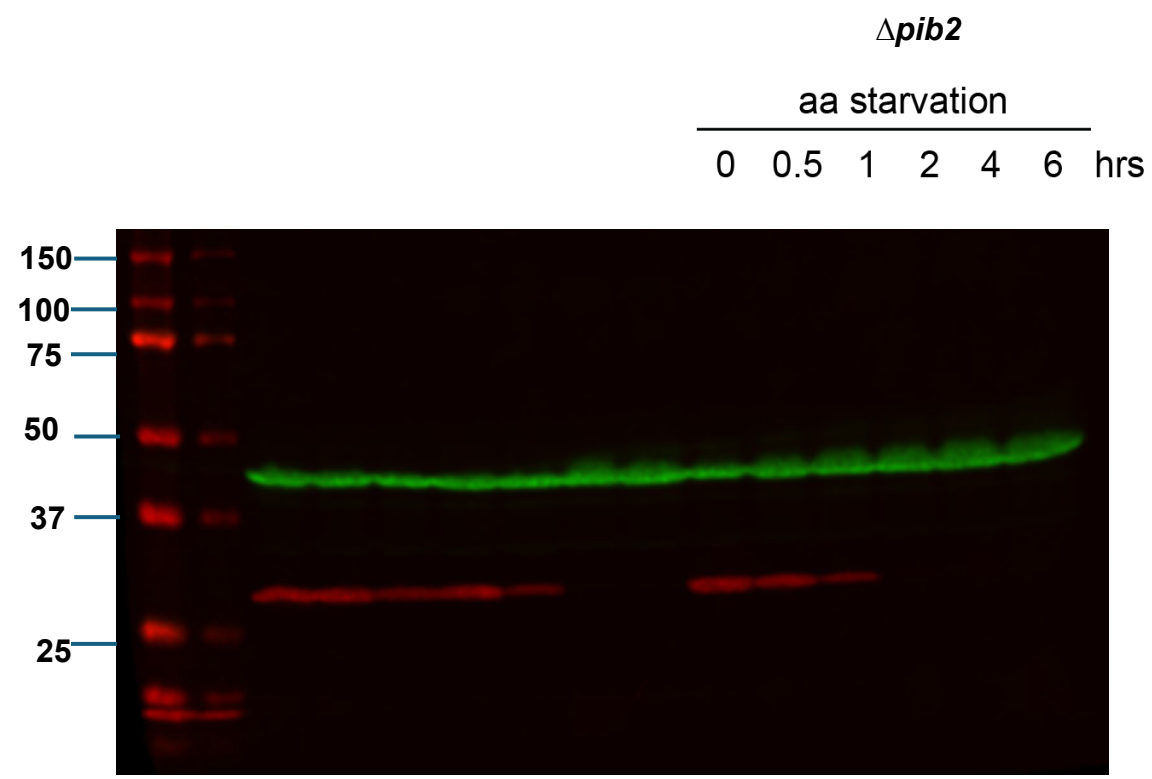

Figure 1B

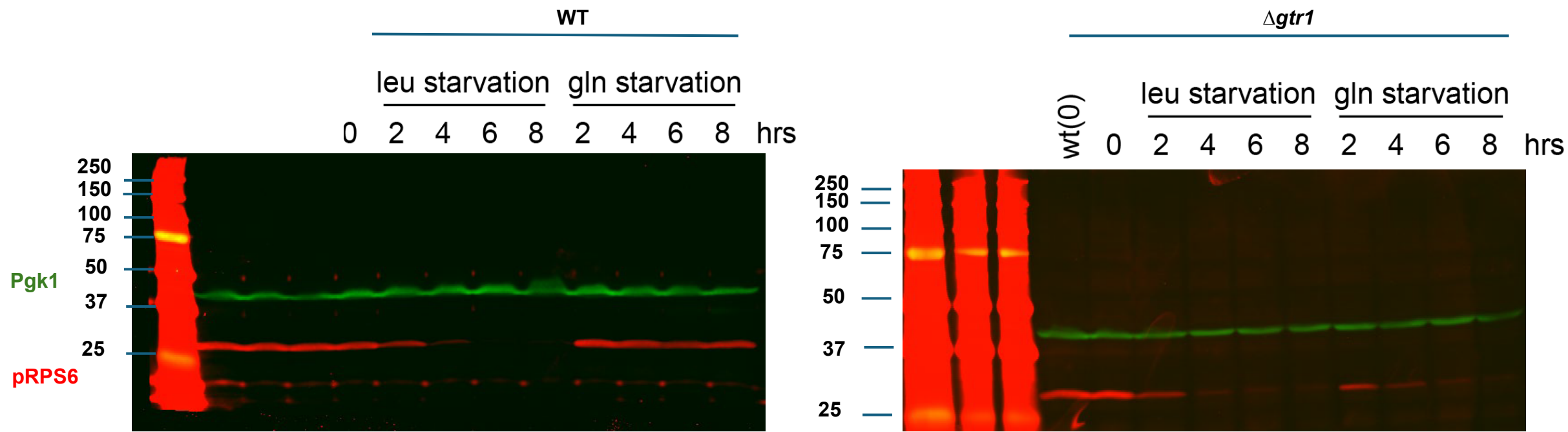

Figure 1B (cont)

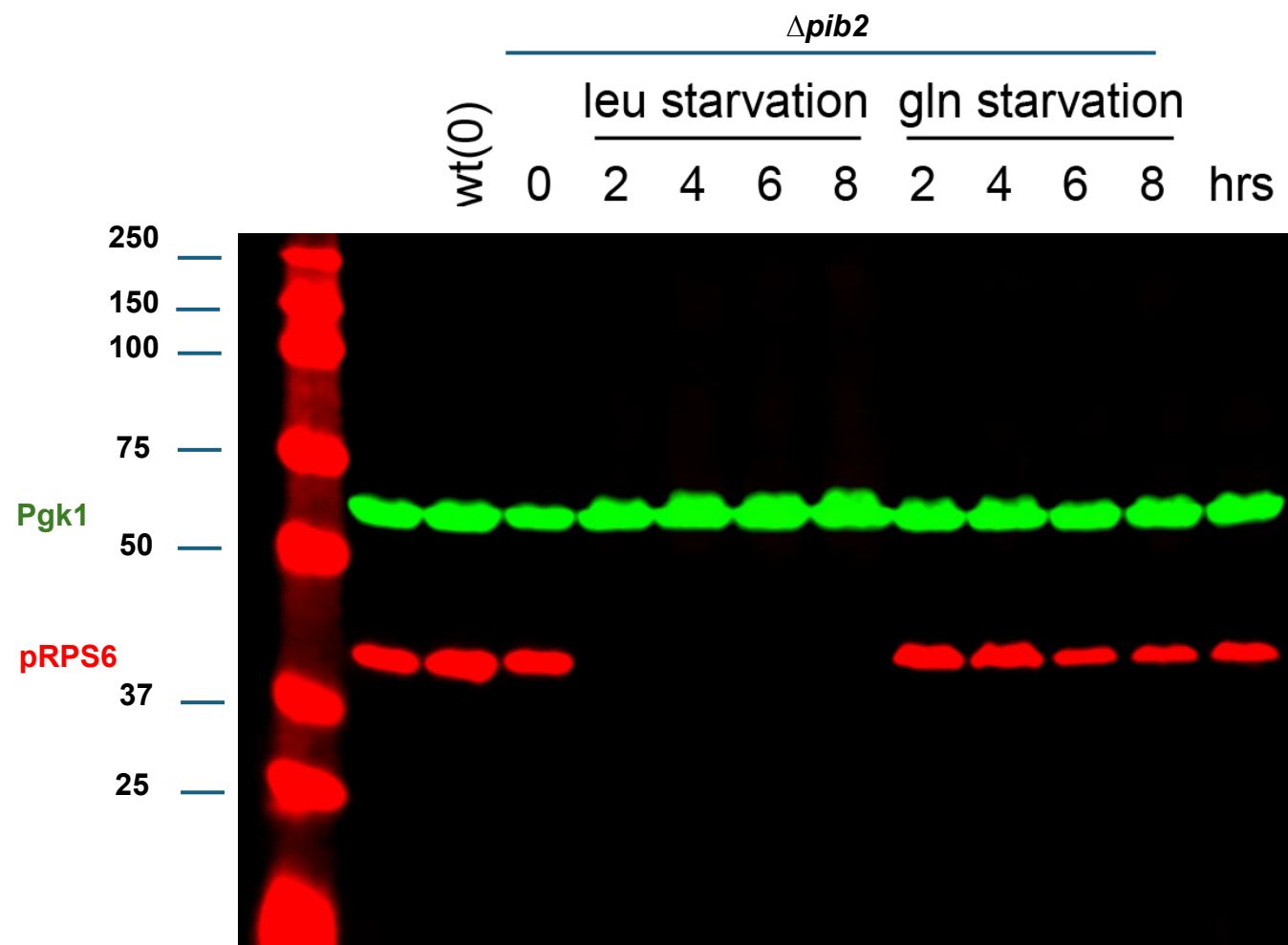

Supplement: Figure 1—source data 1. [file elife-94628-fig1-data1.pdf]

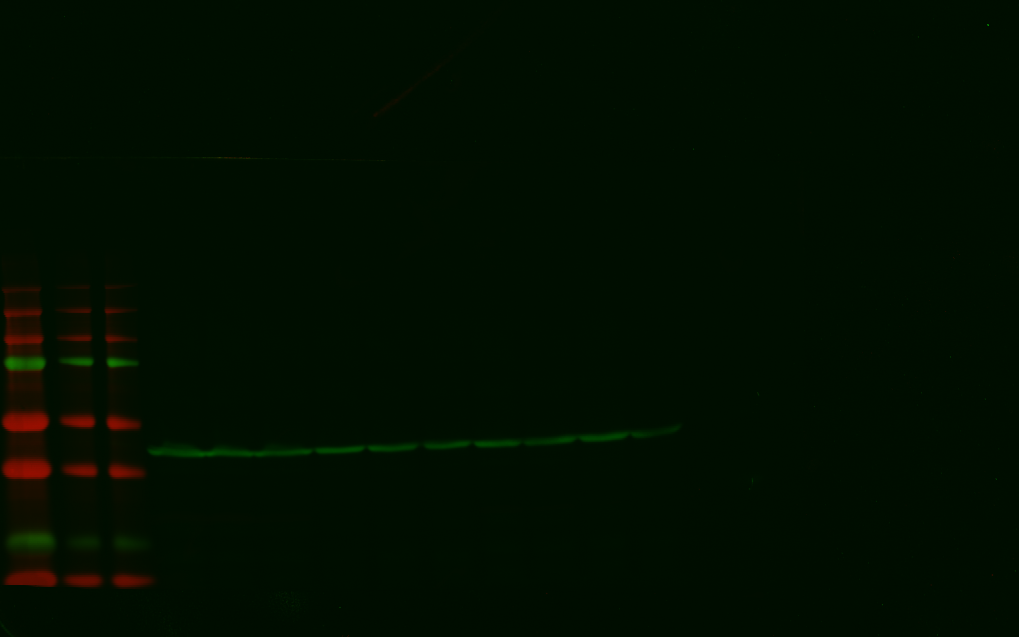

Supplement: Figure 1—source data 2. [file elife-94628-fig1-data2.zip › Figure 1-source data 2/1B_rps6_-leu_-gln_gtr1ko.tif]

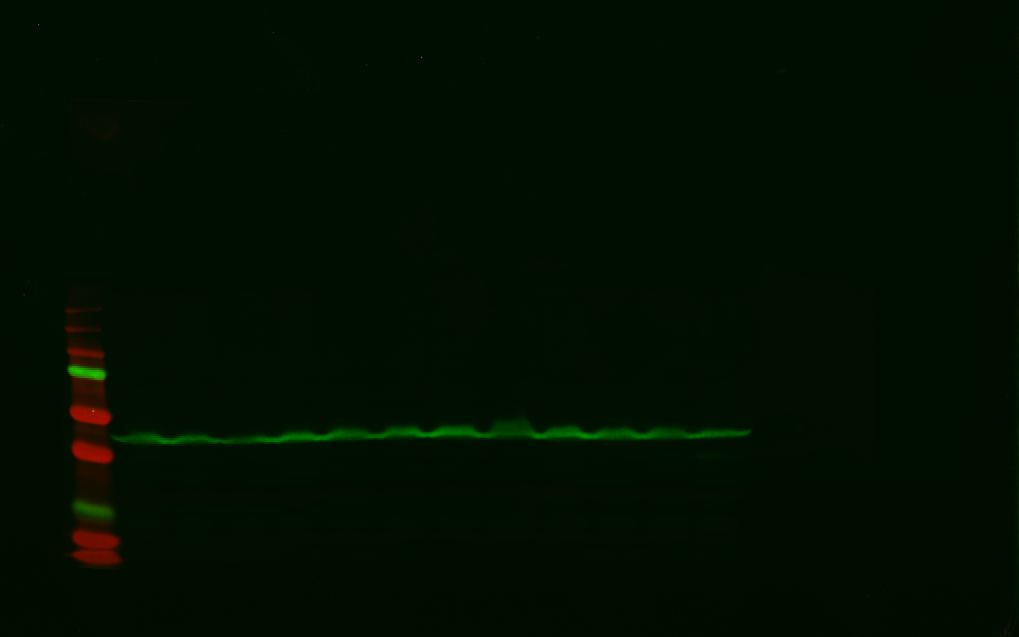

Supplement: Figure 1—source data 2. [file elife-94628-fig1-data2.zip › Figure 1-source data 2/1B_rps6_-leu_-gln_wt.tif]

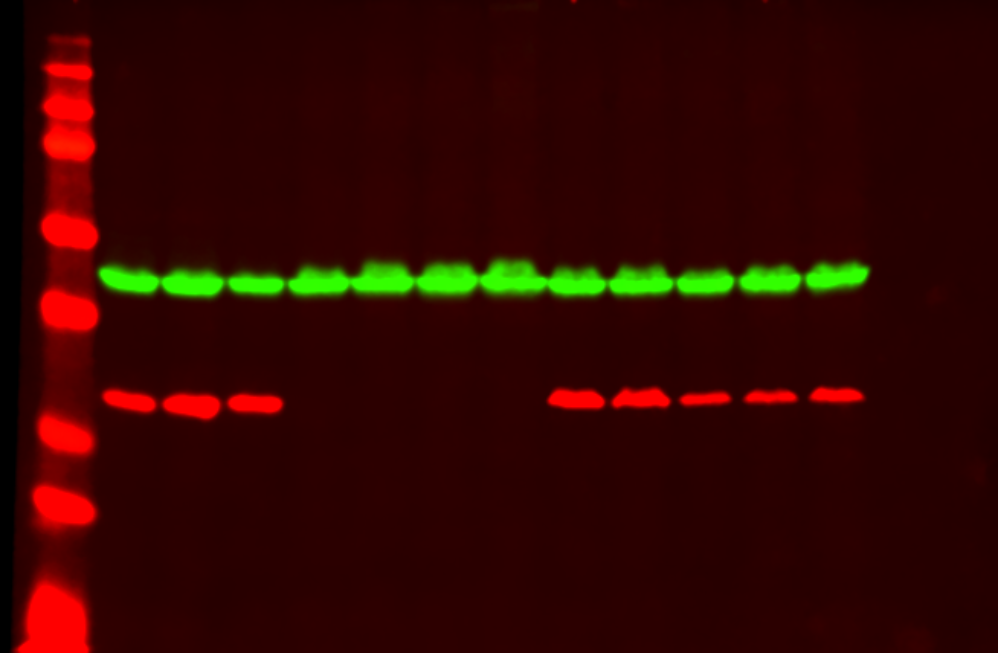

Supplement: Figure 1—source data 2. [file elife-94628-fig1-data2.zip › Figure 1-source data 2/1B_rps6_-leu_-gln_pib2ko.tif]

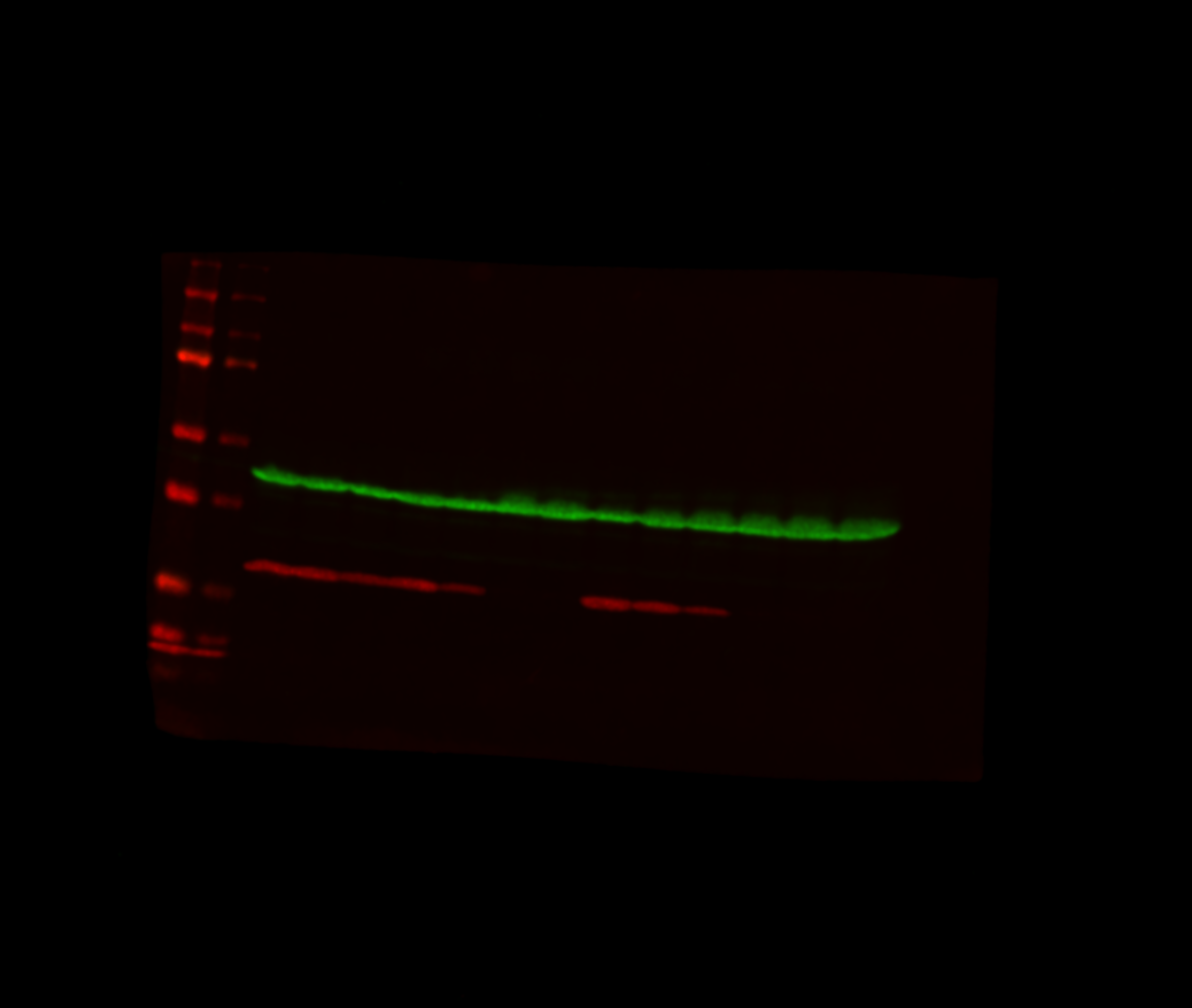

Supplement: Figure 1—source data 2. [file elife-94628-fig1-data2.zip › Figure 1-source data 2/1A_rps6_amino-acids_pib2ko.tif]

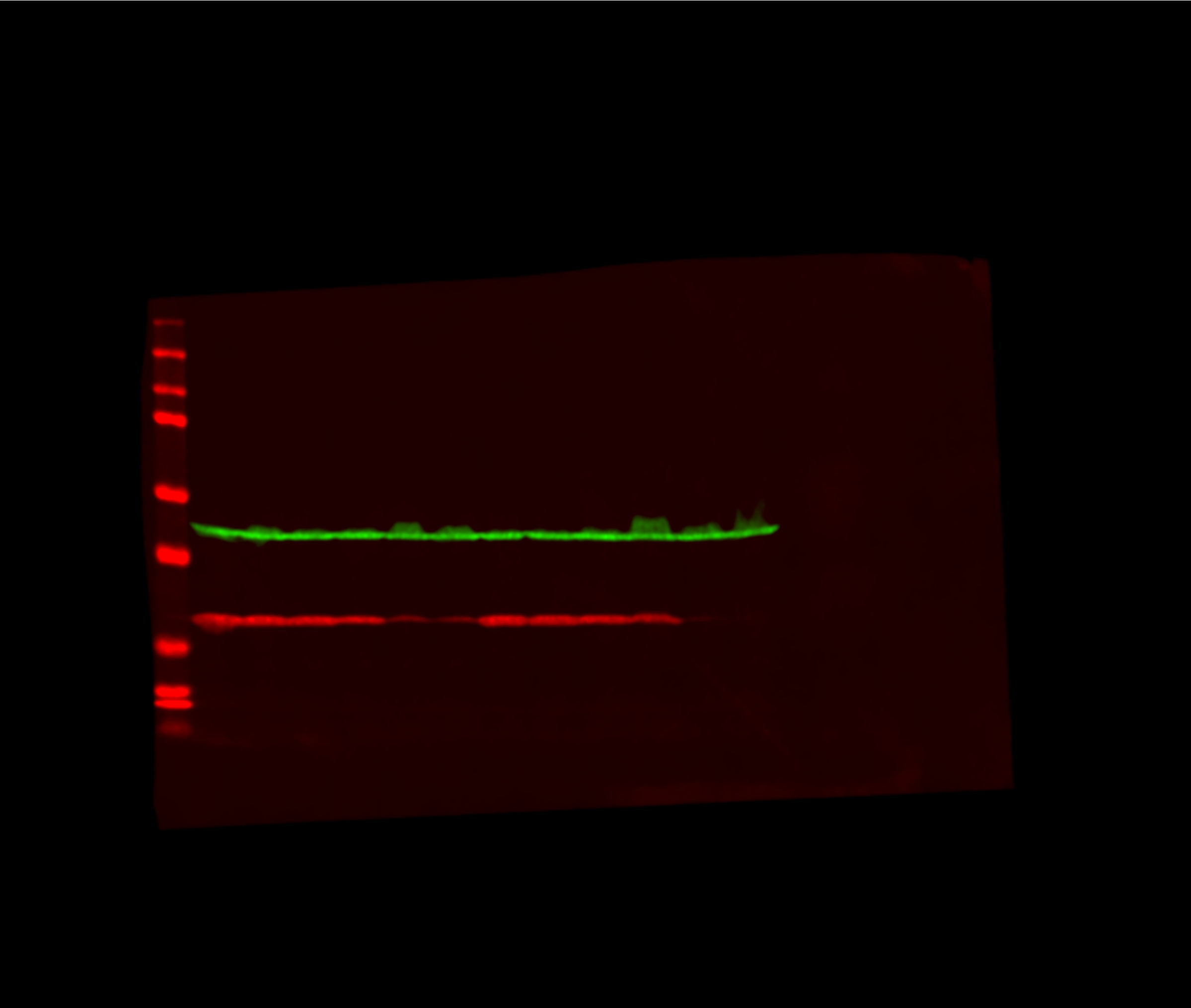

Supplement: Figure 1—source data 2. [file elife-94628-fig1-data2.zip › Figure 1-source data 2/1A_rps6_amino-acids_wt_gtr1ko.tif]

Figure 2A

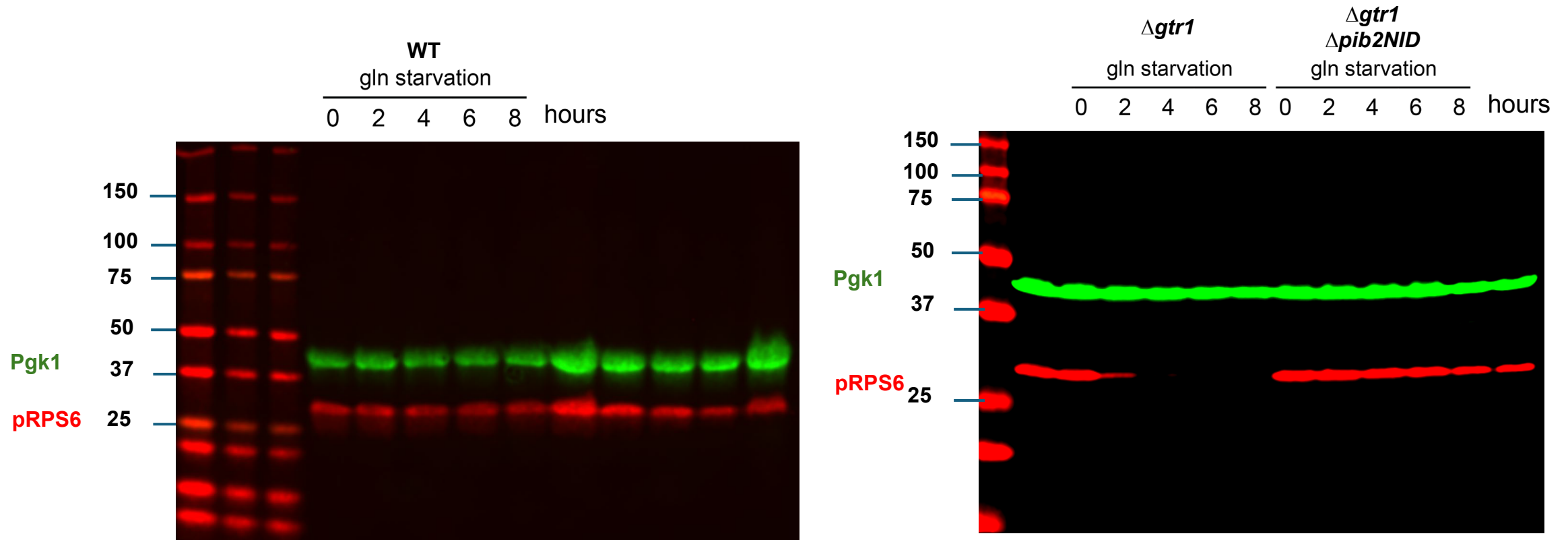

Figure 2A cont

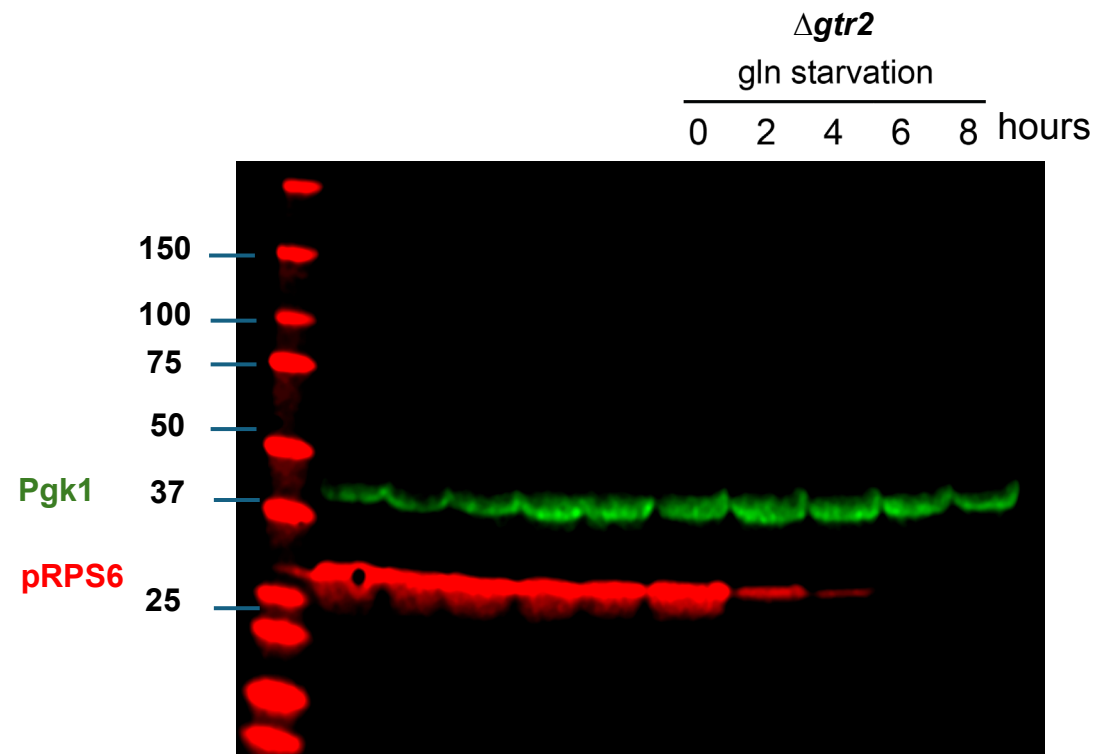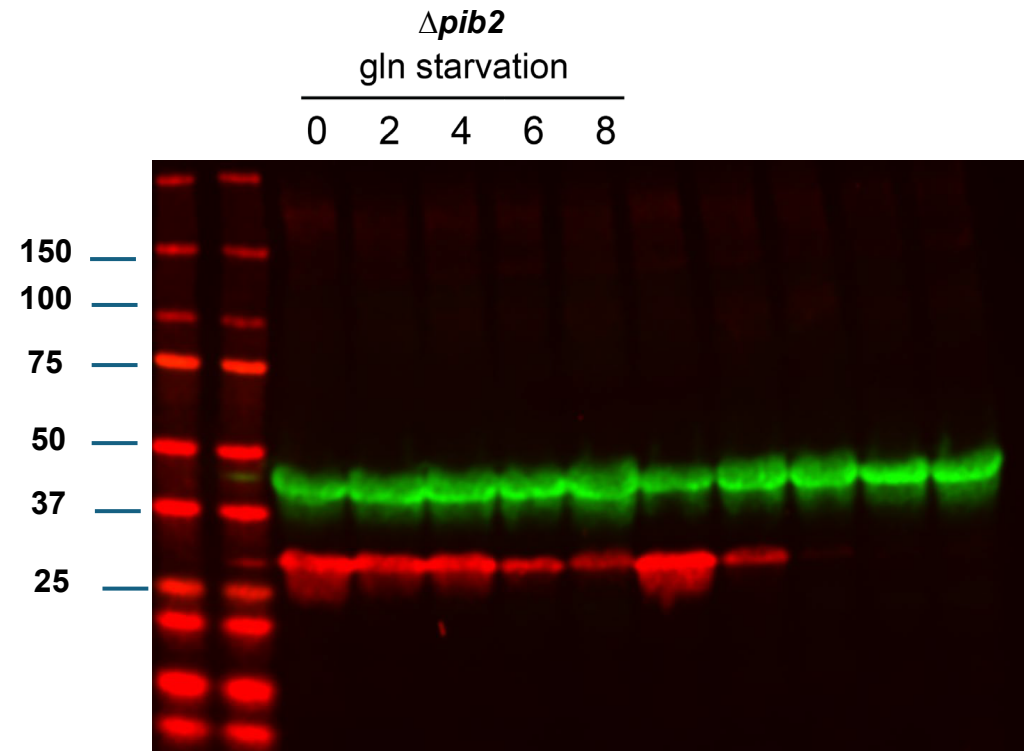

Figure 2A cont

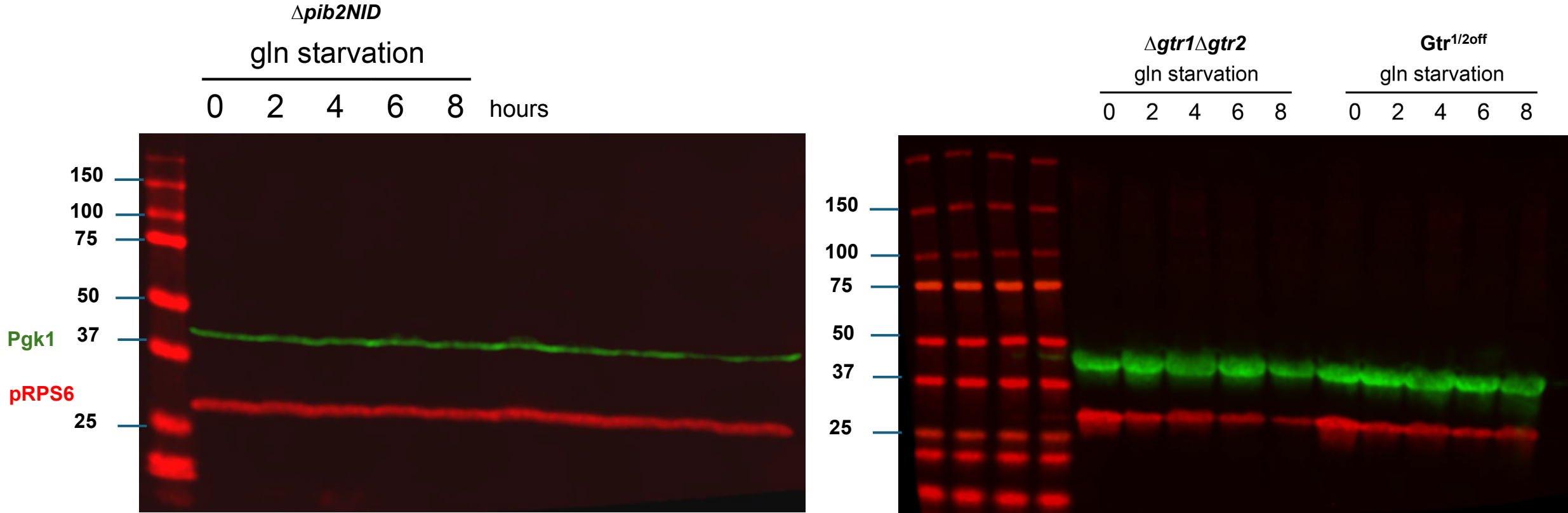

Supplement: Figure 2—source data 1. [file elife-94628-fig2-data1.pdf]

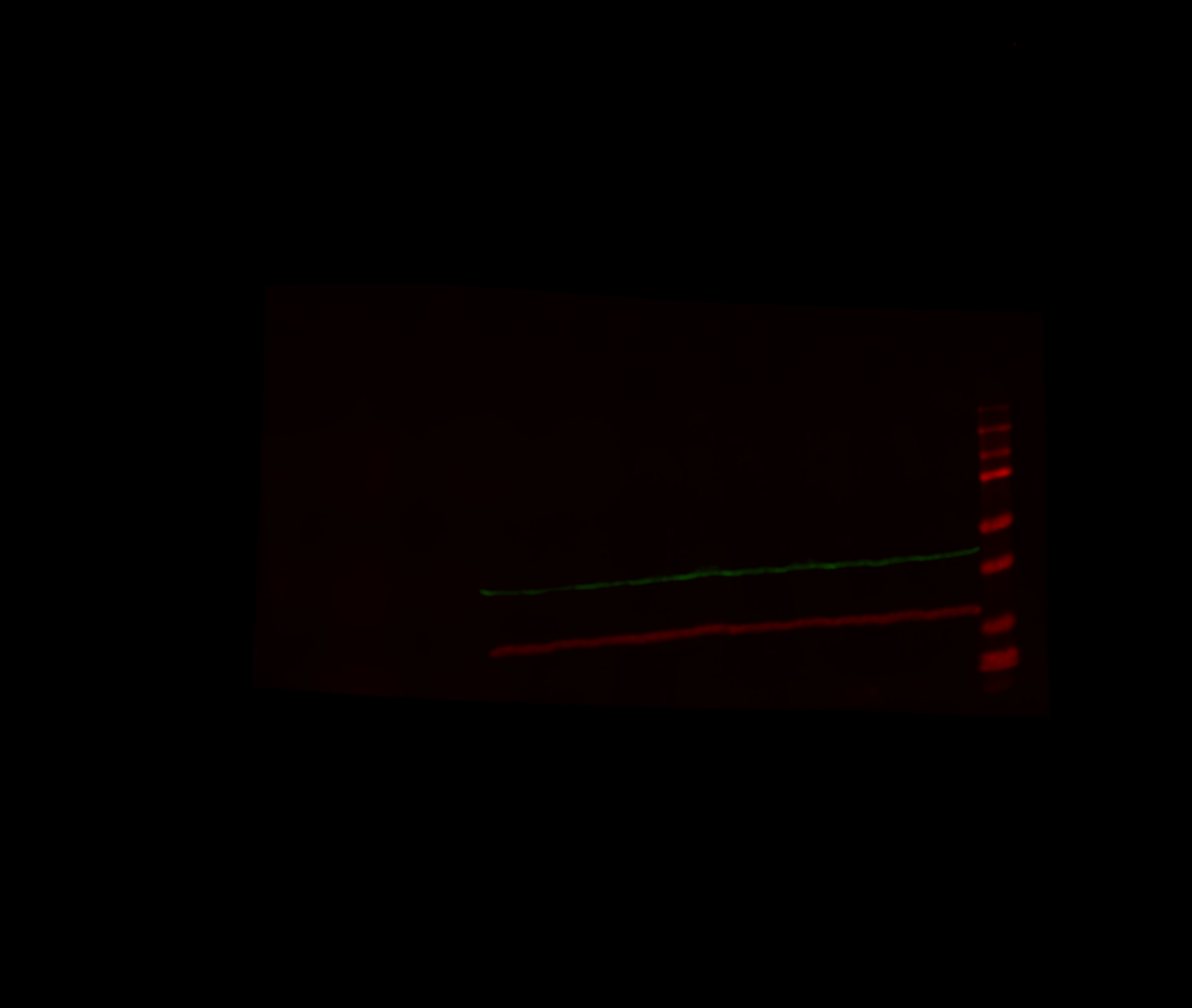

Supplement: Figure 2—source data 2. [file elife-94628-fig2-data2.zip › Figure 2-source data 2/2A_rps6_-gln_pib2nid.tif]

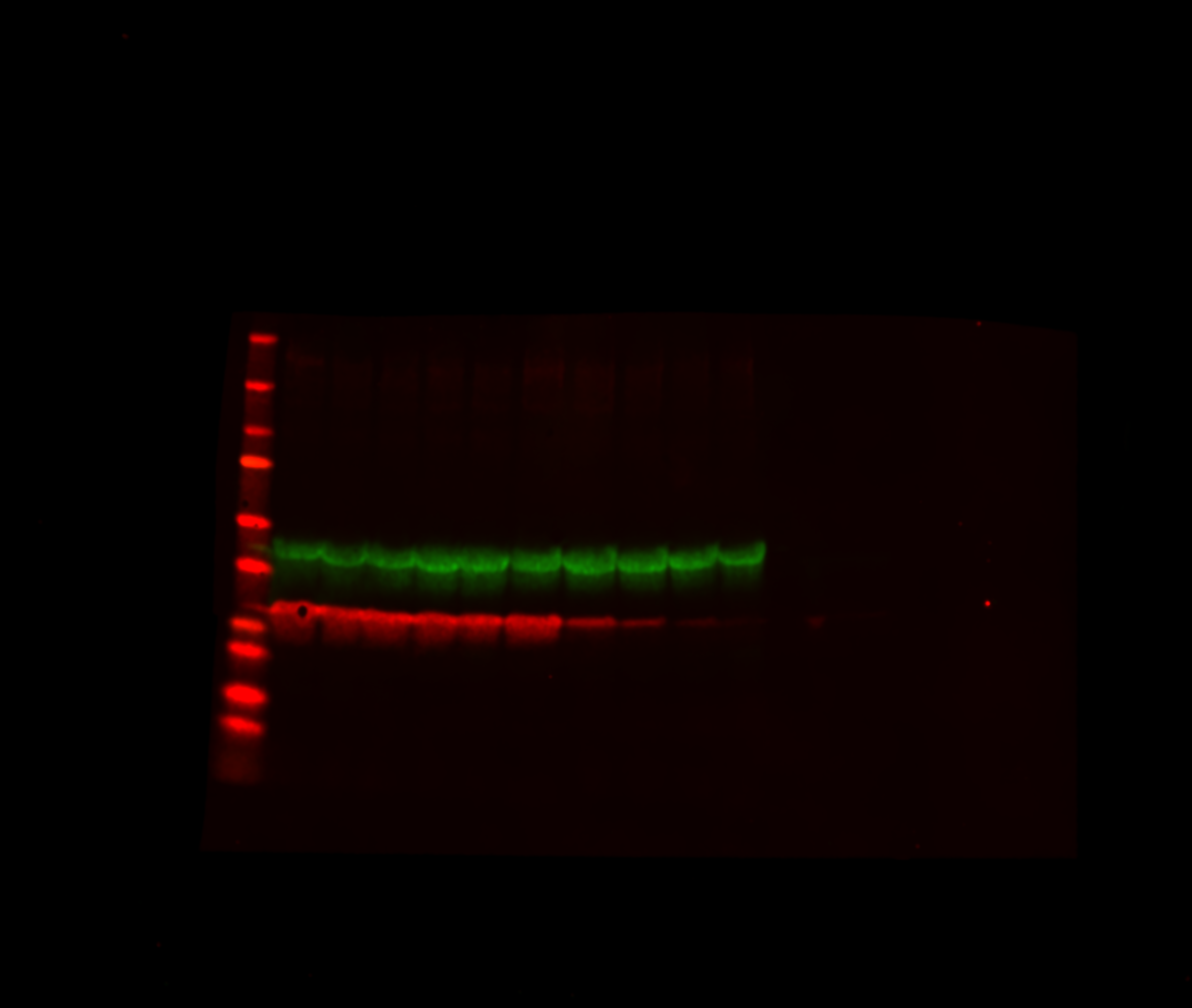

Supplement: Figure 2—source data 2. [file elife-94628-fig2-data2.zip › Figure 2-source data 2/2A_rps6_-gln_gtr2ko.tif]

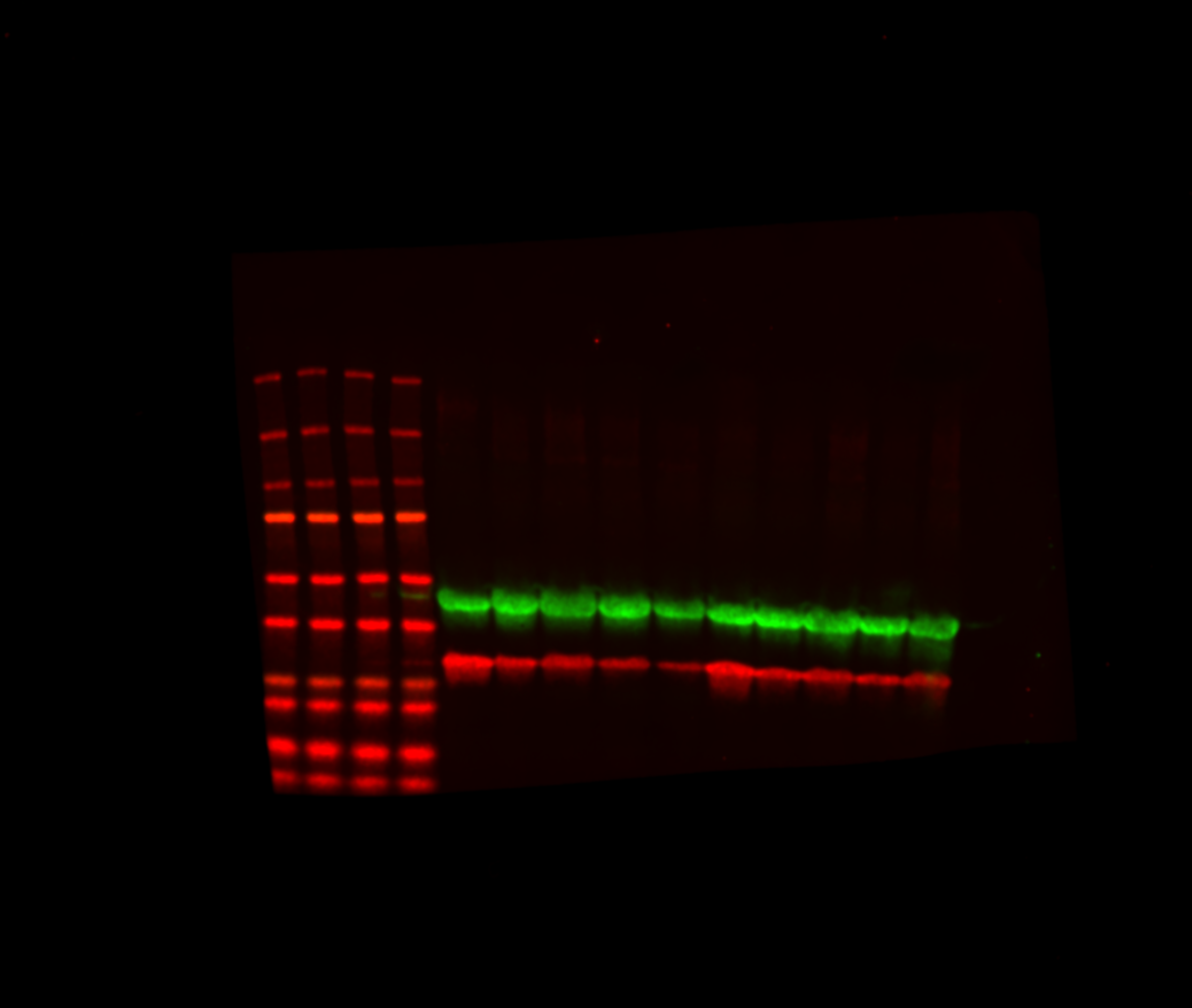

Supplement: Figure 2—source data 2. [file elife-94628-fig2-data2.zip › Figure 2-source data 2/2A_rps6_-gln_gtr1-2ko_gtr1-2off.tif]

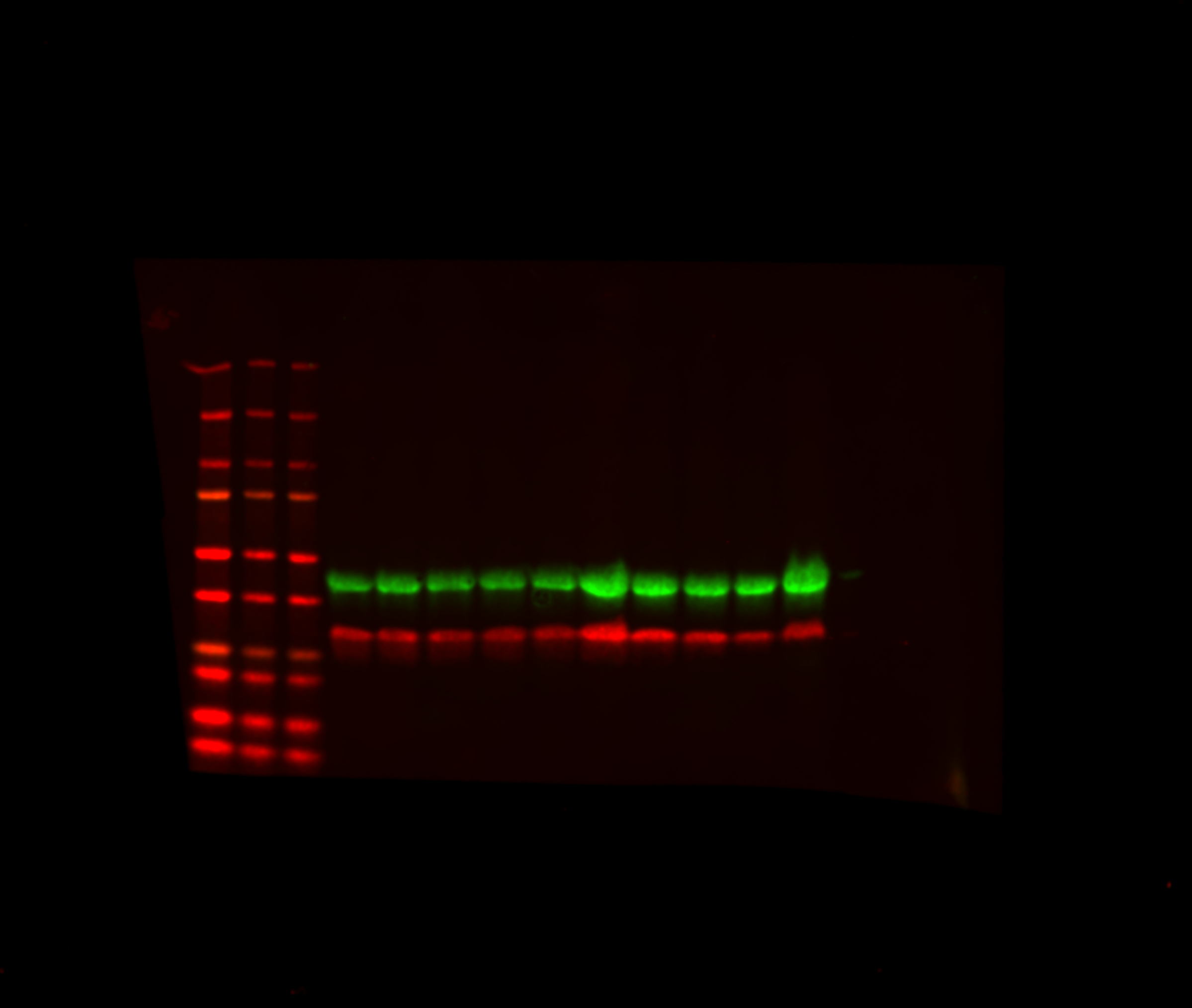

Supplement: Figure 2—source data 2. [file elife-94628-fig2-data2.zip › Figure 2-source data 2/2A_rps6_-gln_WT.tif]

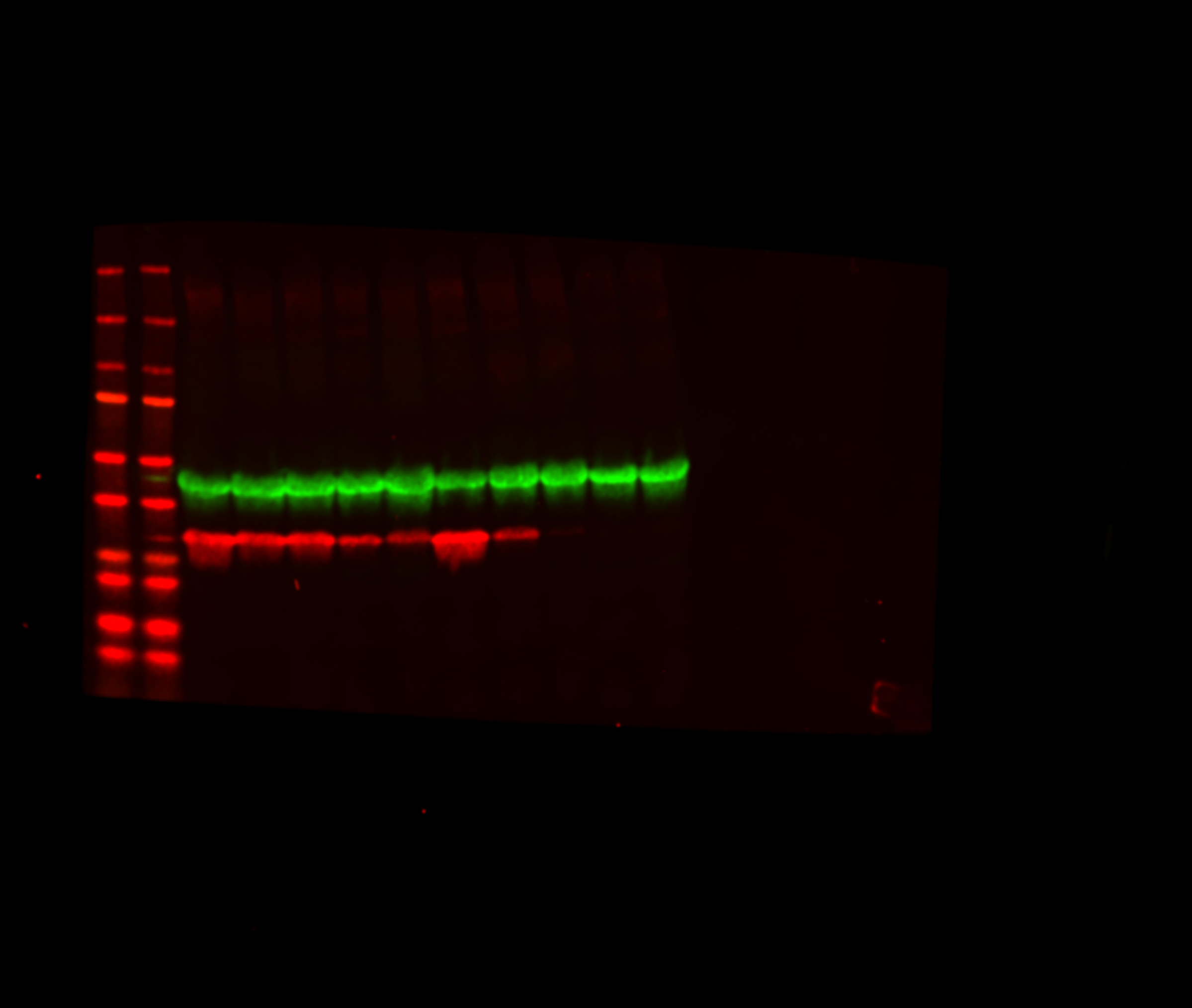

Supplement: Figure 2—source data 2. [file elife-94628-fig2-data2.zip › Figure 2-source data 2/2A_rps6_-gln_pib2ko.tif]

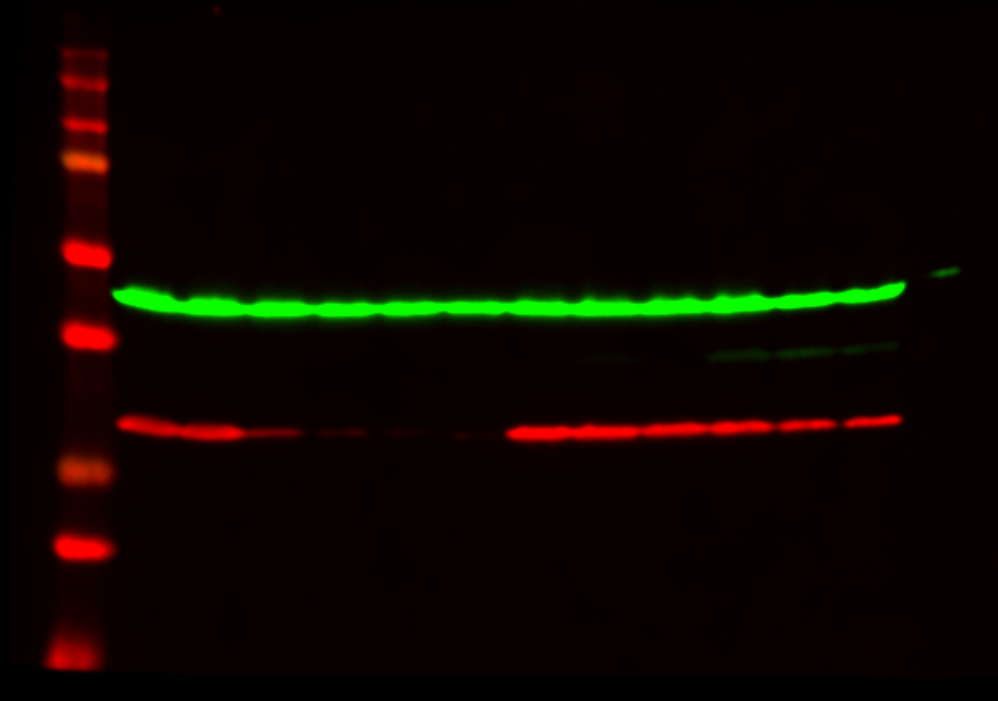

Supplement: Figure 2—source data 2. [file elife-94628-fig2-data2.zip › Figure 2-source data 2/2A_rps6_-gln_gtr1ko_gtr1ko-pib2nid.tif]

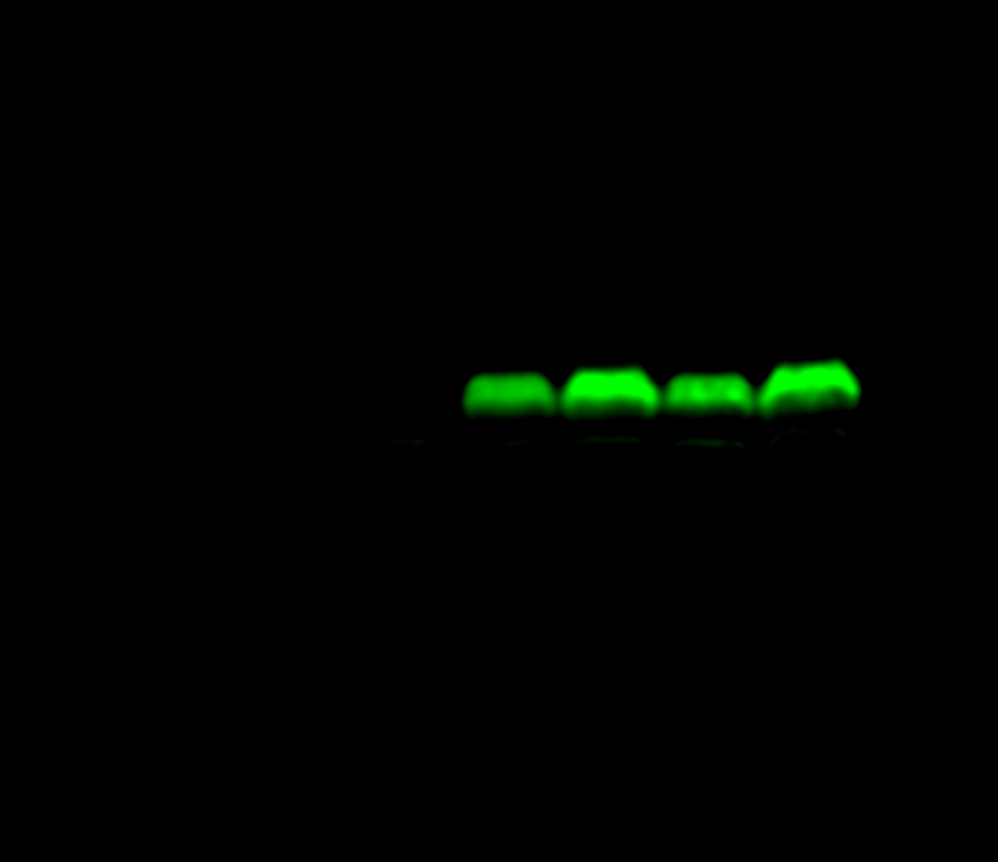

Supplement: Figure 5—source data 2. [file elife-94628-fig5-data2.zip › Figure 5-source data 2/5G_Gtr1-Myc_HA-Ser33_co-IP_Ser33_Input.tif]

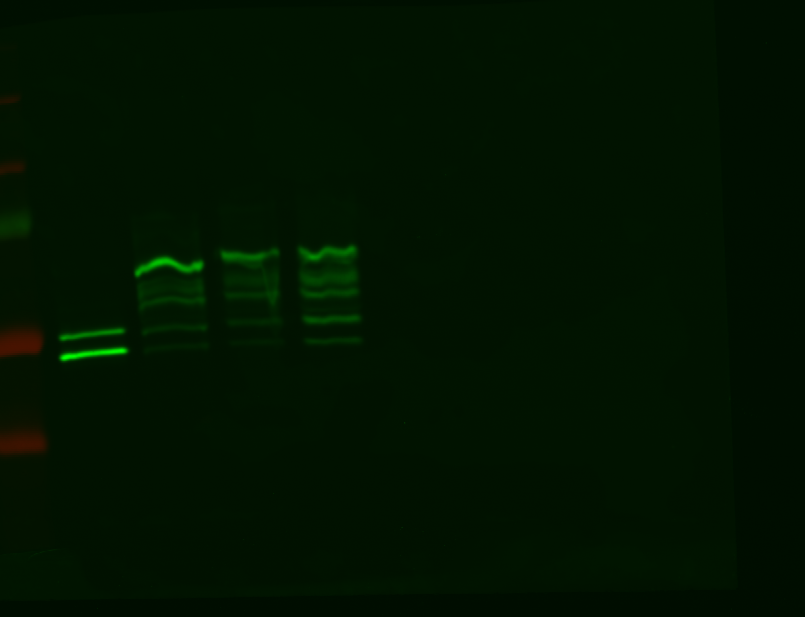

Supplement: Figure 5—source data 2. [file elife-94628-fig5-data2.zip › Figure 5-source data 2/5F_Ser33_PT_gel_pro-gln_upshift.tif]

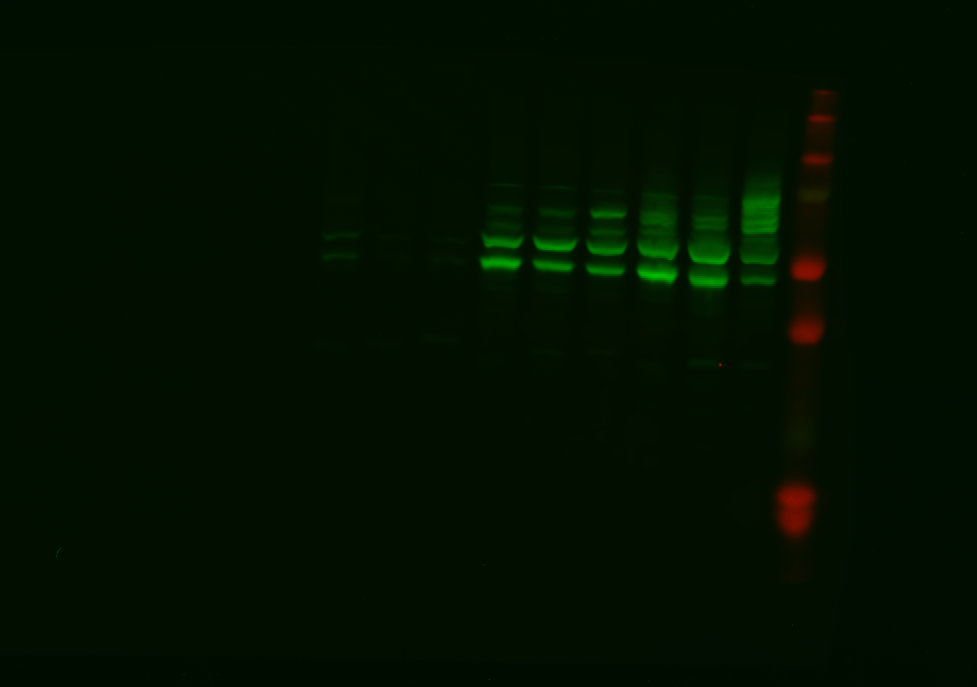

Supplement: Figure 5—source data 2. [file elife-94628-fig5-data2.zip › Figure 5-source data 2/5C_Ser33_PT_gel_rap_-N.tif]

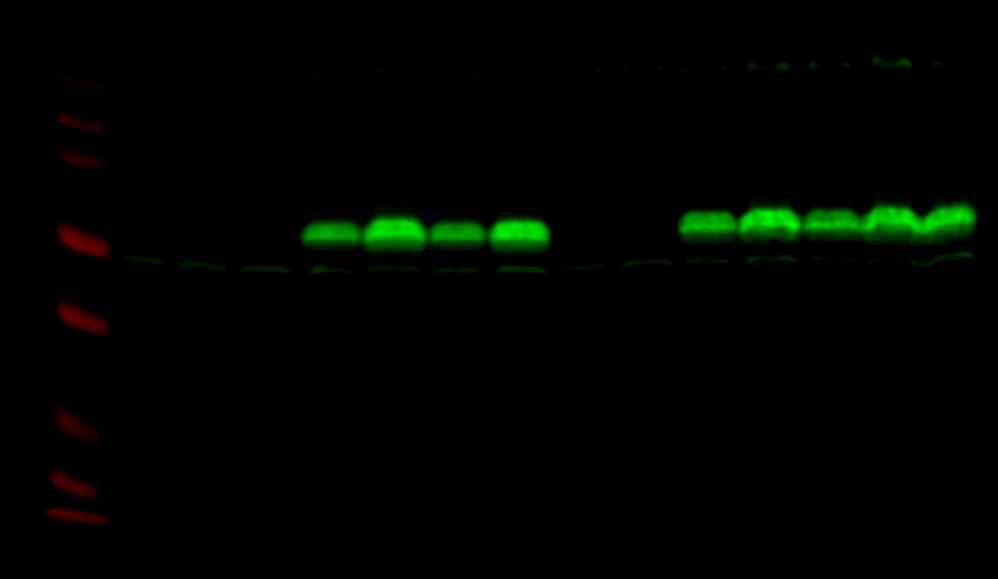

Supplement: Figure 5—source data 2. [file elife-94628-fig5-data2.zip › Figure 5-source data 2/5G_Gtr1-Myc_HA-Ser33_co-IP_INPUT_left-side.tif]

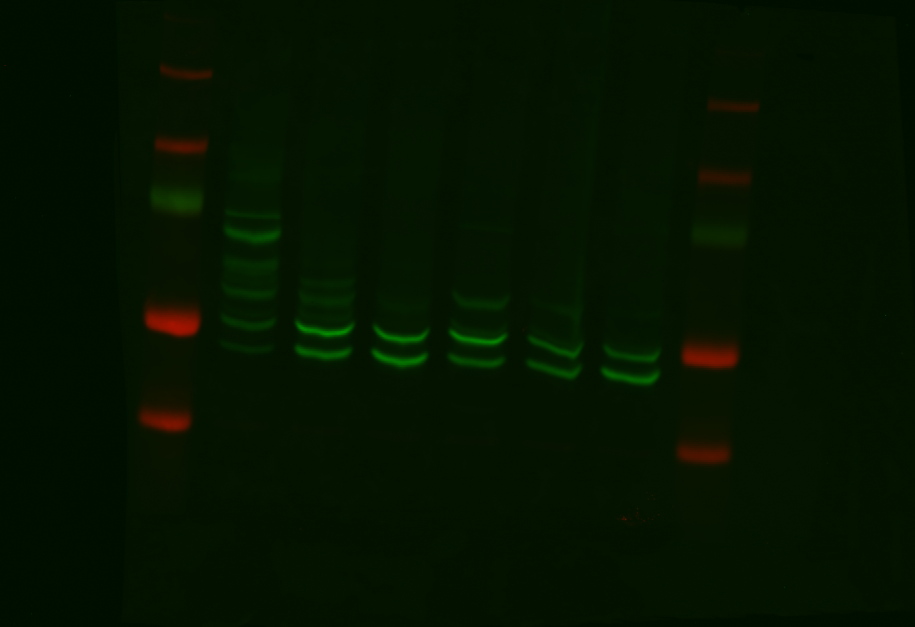

Supplement: Figure 5—source data 2. [file elife-94628-fig5-data2.zip › Figure 5-source data 2/5C_Ser33_PT_gel_SL.tif]

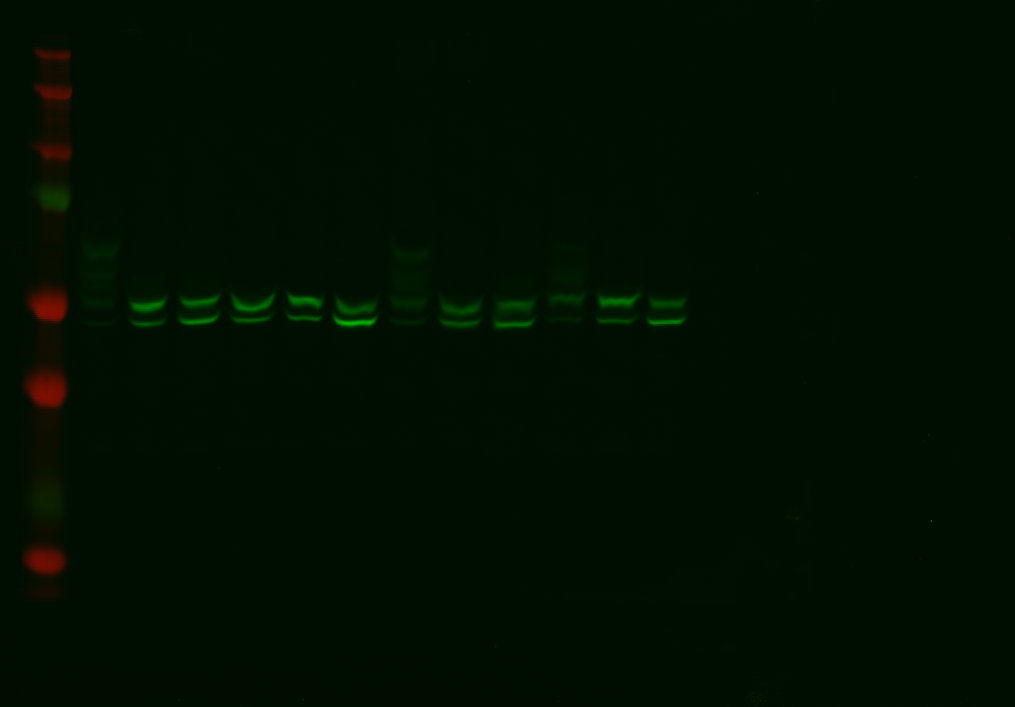

Supplement: Figure 5—source data 2. [file elife-94628-fig5-data2.zip › Figure 5-source data 2/5D_Ser33_PT_gel_Pib2-mutants.tif]

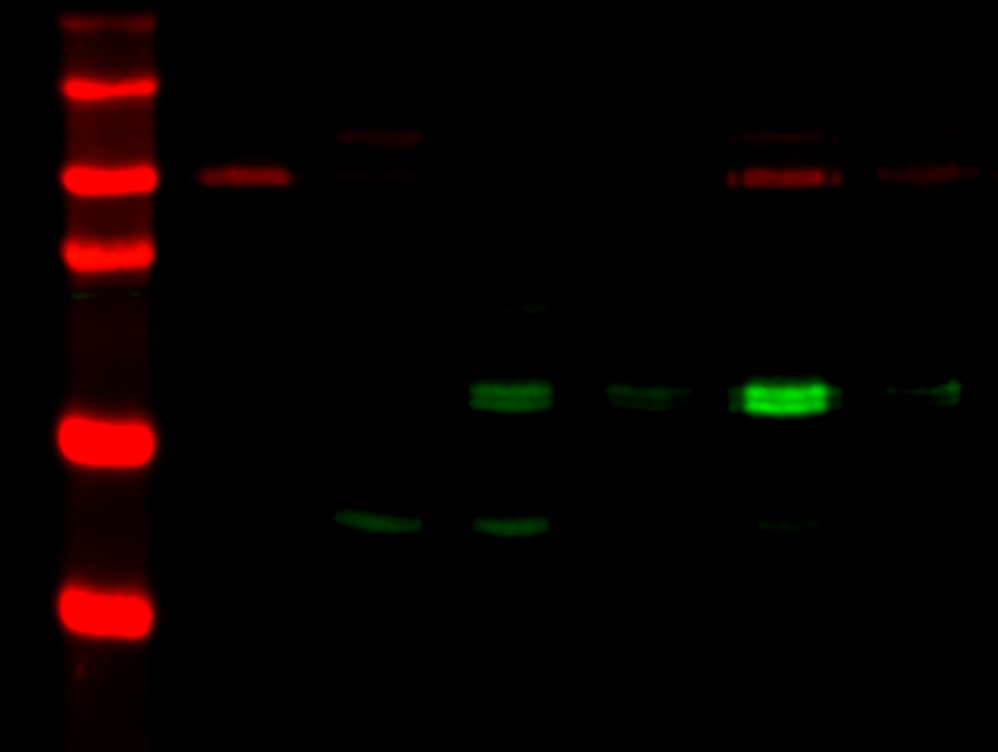

Supplement: Figure 5—source data 2. [file elife-94628-fig5-data2.zip › Figure 5-source data 2/GFP-Pib2_HA-Ser33_coIP.tif]

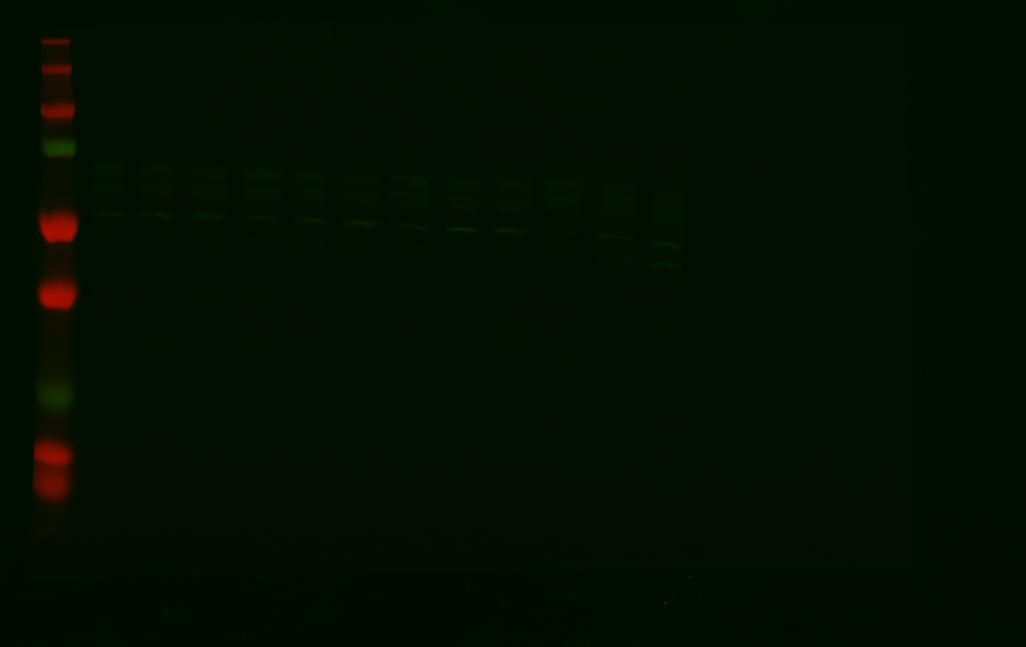

Supplement: Figure 5—source data 2. [file elife-94628-fig5-data2.zip › Figure 5-source data 2/5E_Ser33_PT_-gln_-leu.tif]

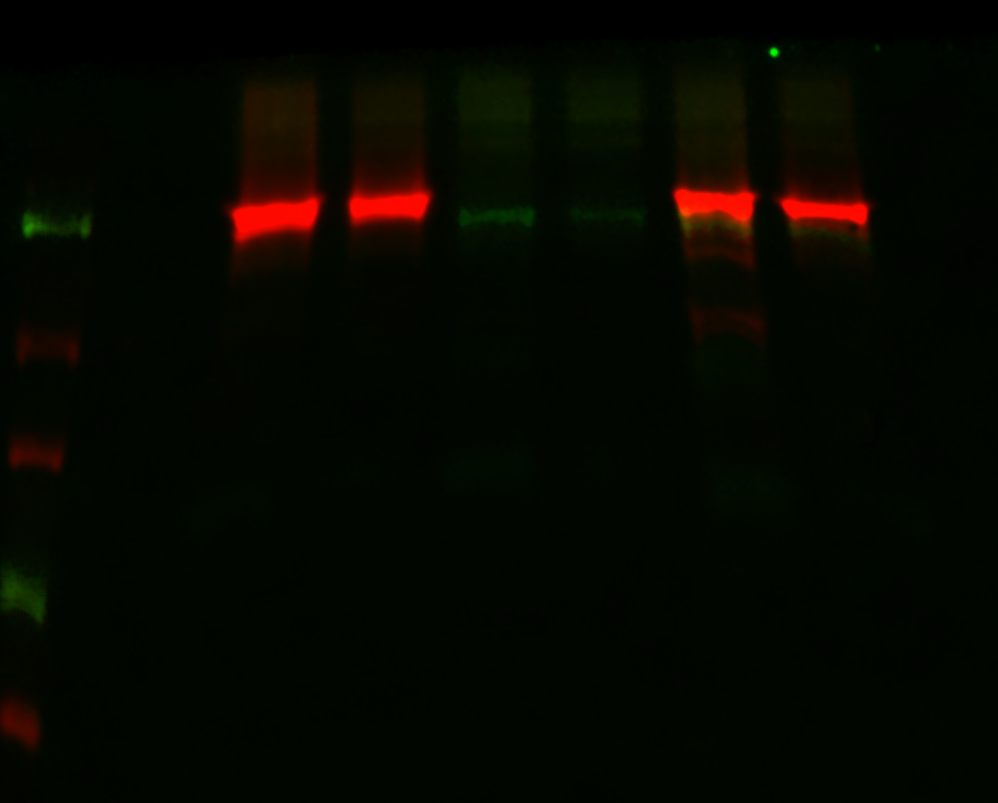

Supplement: Figure 5—source data 2. [file elife-94628-fig5-data2.zip › Figure 5-source data 2/5G_Gtr1-Myc_HA-Ser33_co-IP.tif]

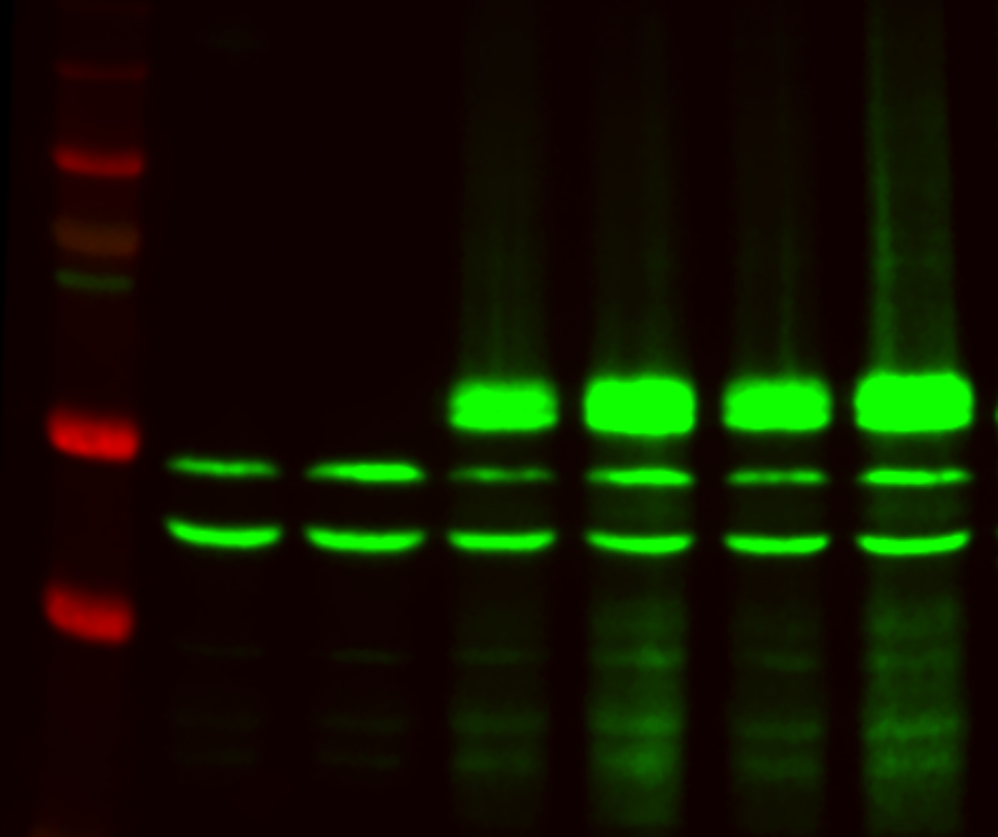

Supplement: Figure 5—source data 2. [file elife-94628-fig5-data2.zip › Figure 5-source data 2/GFP-Pib2_HA-Ser33_coIP_Ser33-Input.tif]

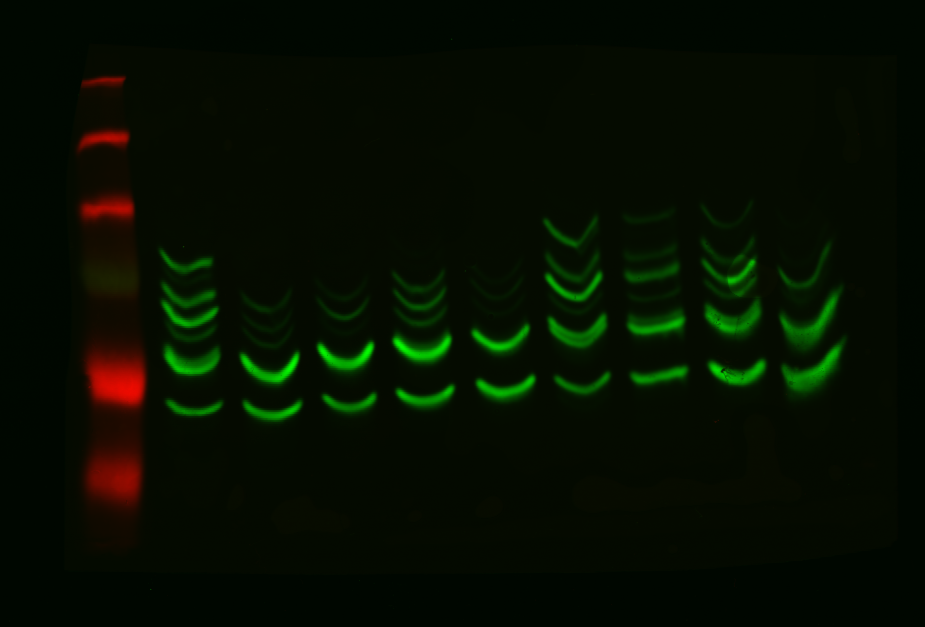

Supplement: Figure 5—source data 2. [file elife-94628-fig5-data2.zip › Figure 5-source data 2/5B_Ser33-IP_PT_gel.tif]

Figure 5-figure supplement 1a

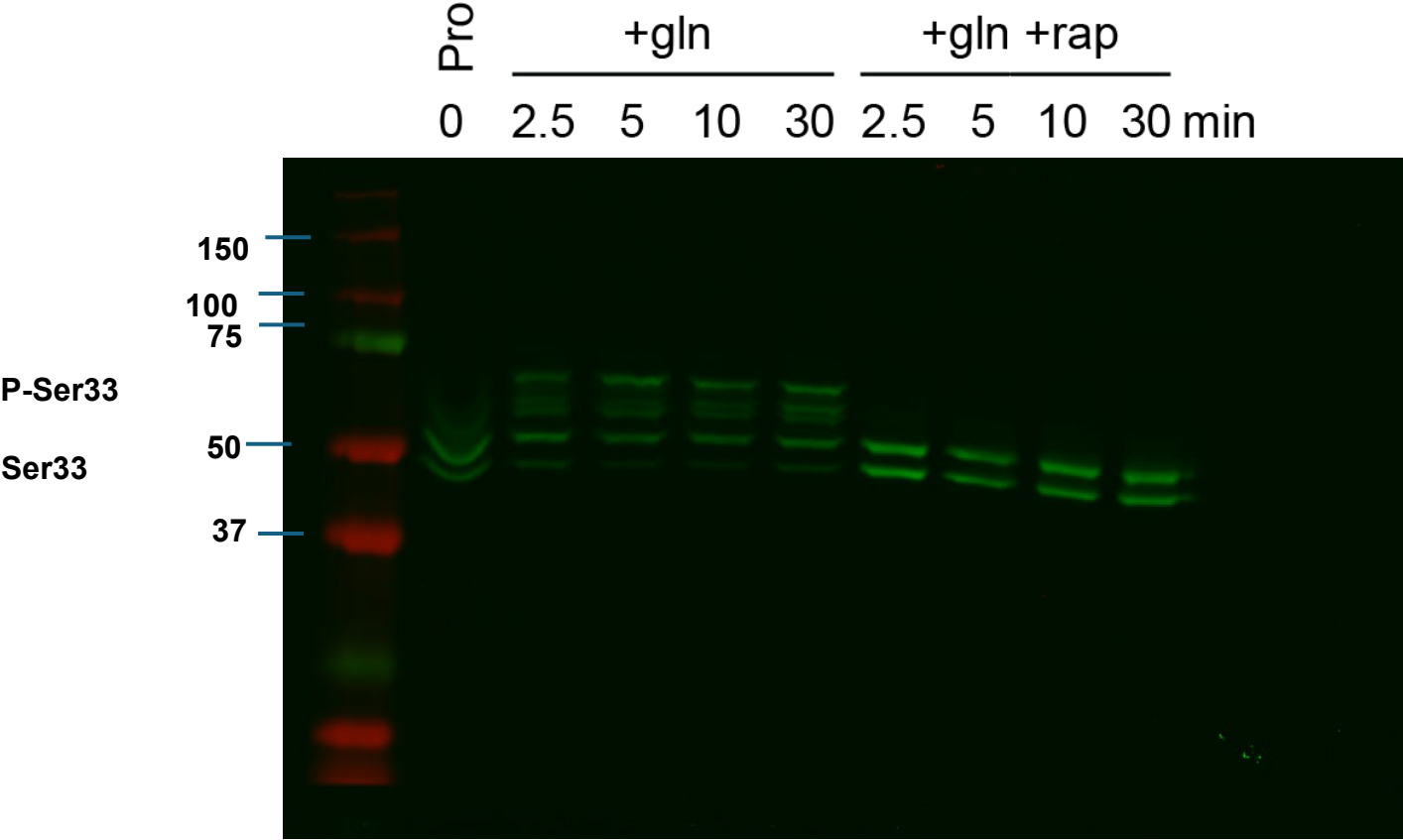

Figure 5-figure supplement 1b

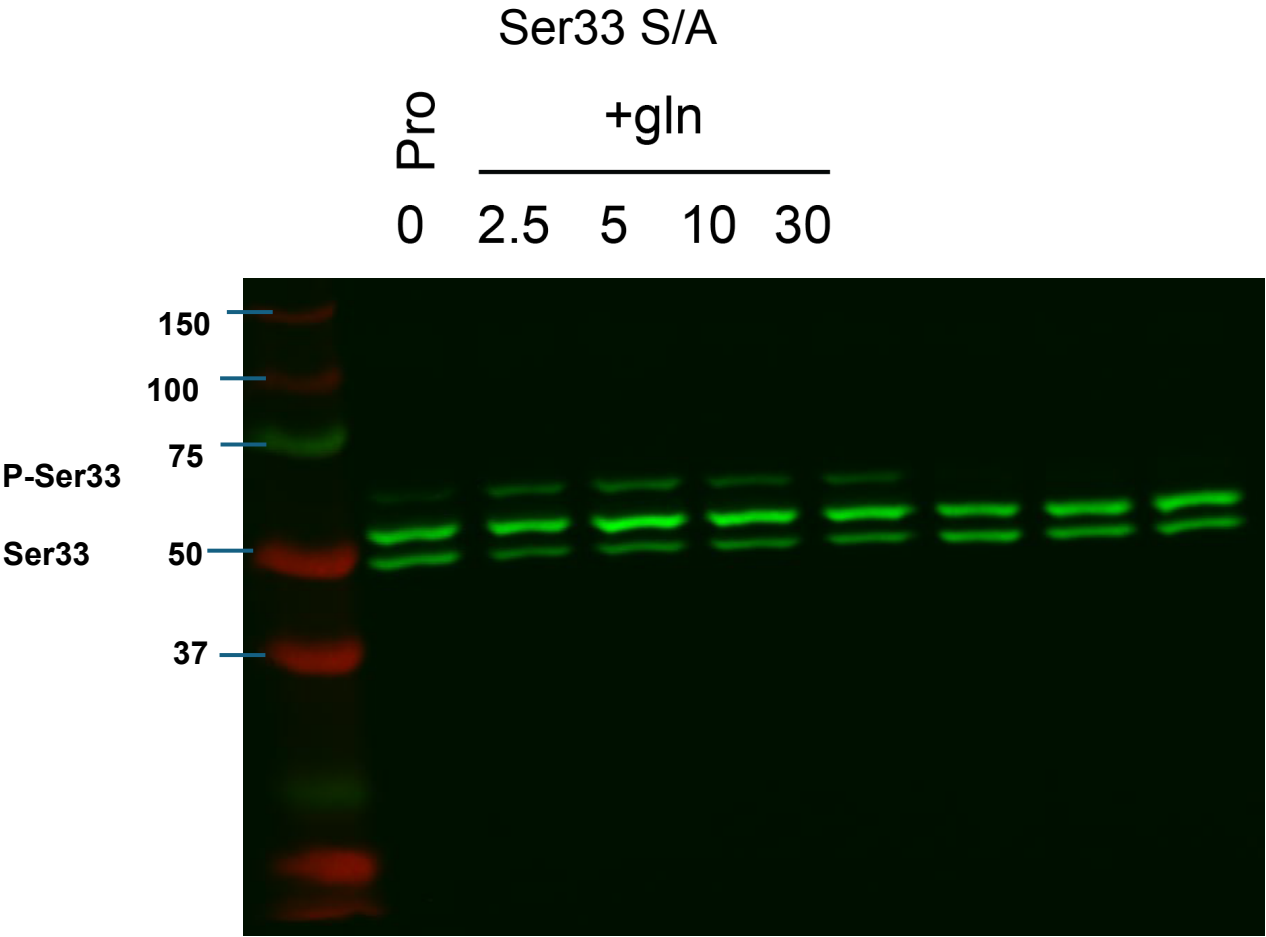

Figure 5-figure supplement 1c

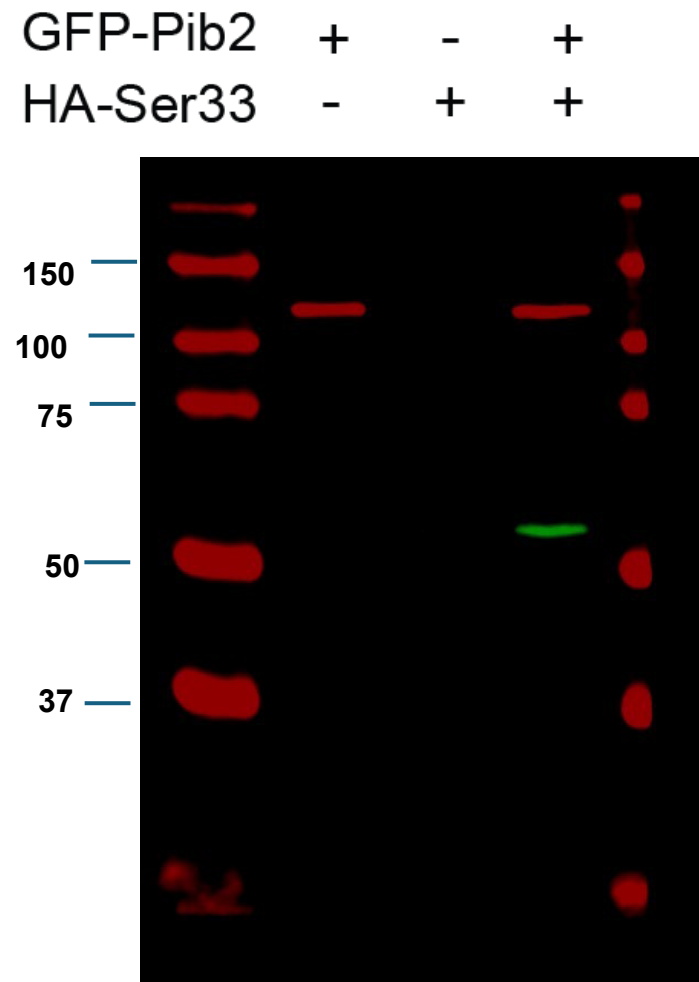

Supplement: Figure 5—figure supplement 1—source data 1. [file elife-94628-fig5-figsupp1-data1.pdf]

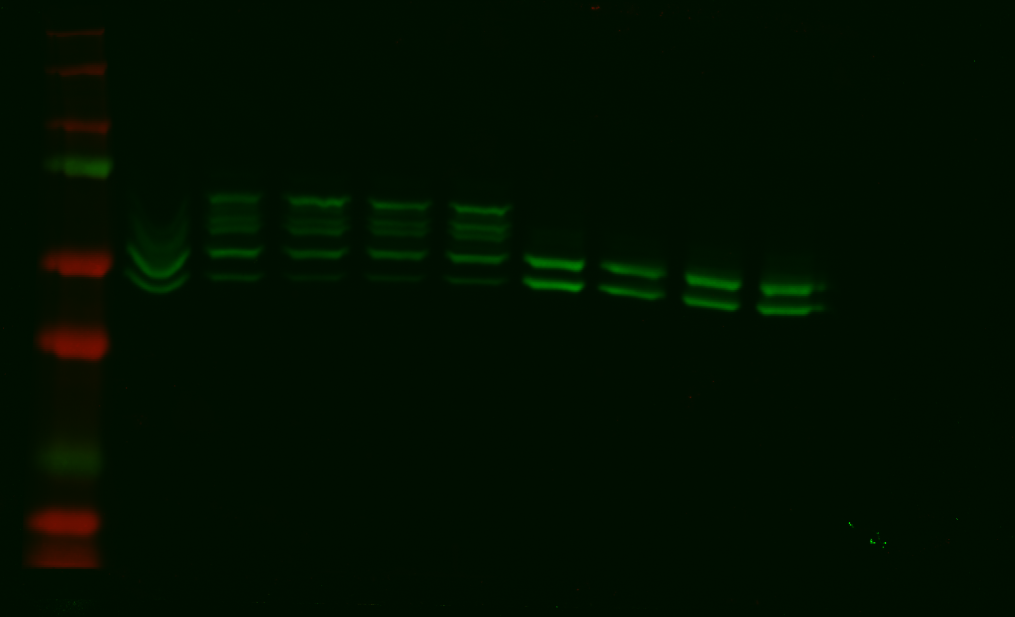

Supplement: Figure 5—figure supplement 1—source data 2. [file elife-94628-fig5-figsupp1-data2.zip › Figure 5-figure supplement 1-source data 2/5-supplement_ser33_pro-gln-shift_WT.tif]

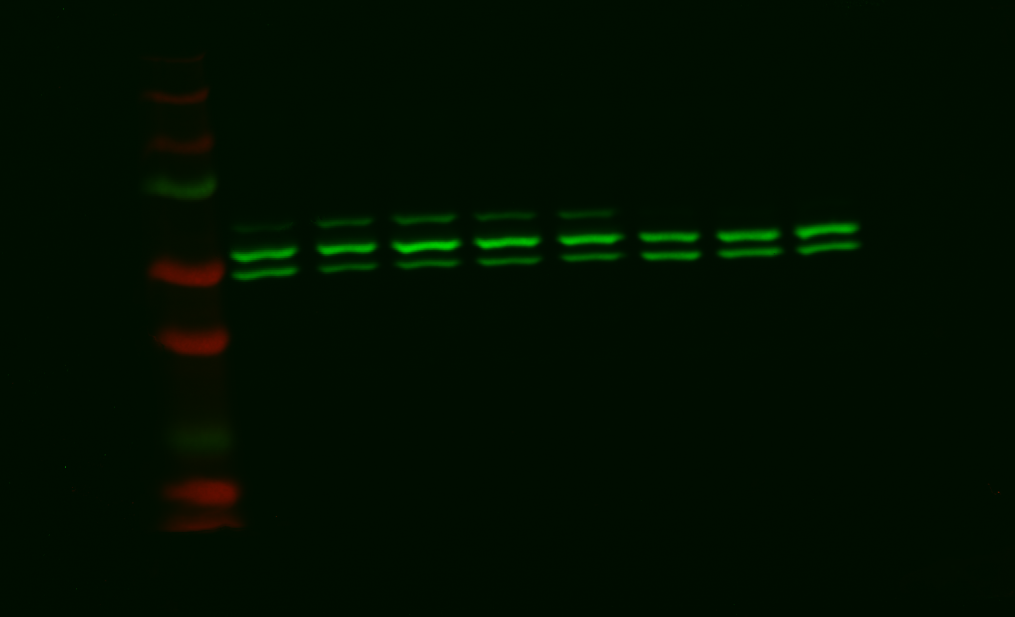

Supplement: Figure 5—figure supplement 1—source data 2. [file elife-94628-fig5-figsupp1-data2.zip › Figure 5-figure supplement 1-source data 2/5-supplement_ser33_pro-gln_shift_S7A.tif]

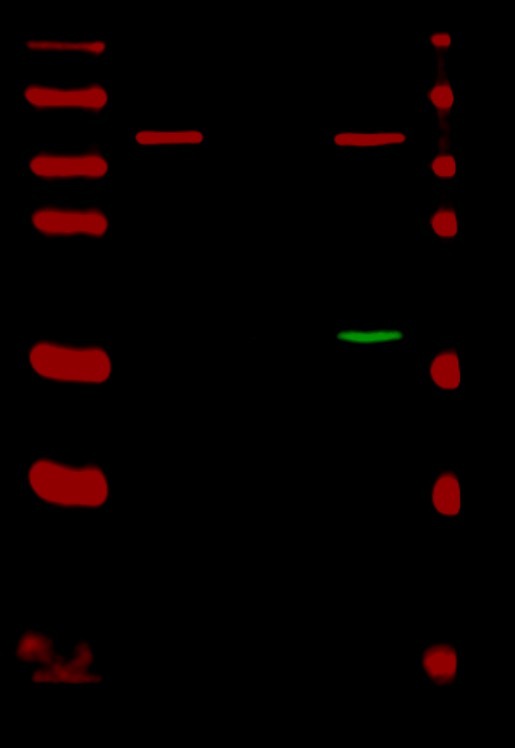

Supplement: Figure 5—figure supplement 1—source data 2. [file elife-94628-fig5-figsupp1-data2.zip › Figure 5-figure supplement 1-source data 2/5-supplement_ser33_pib2-coIP.tif]

Figure 6B

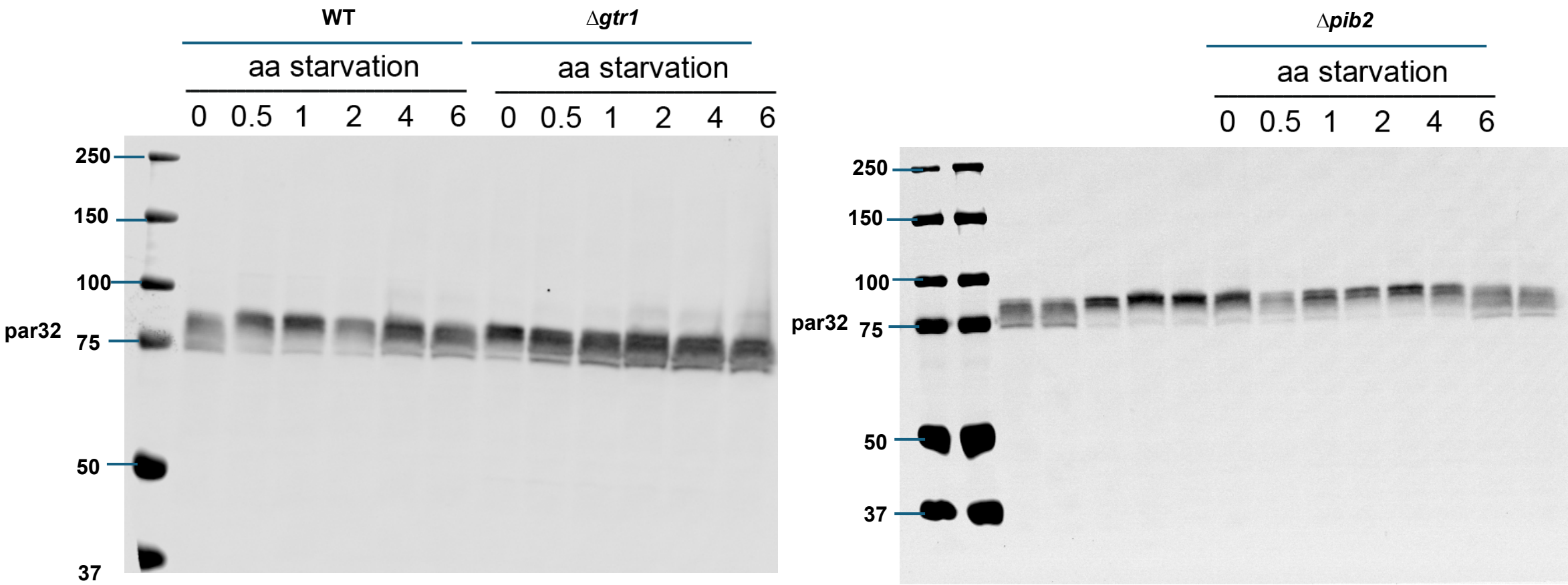

Figure 6C

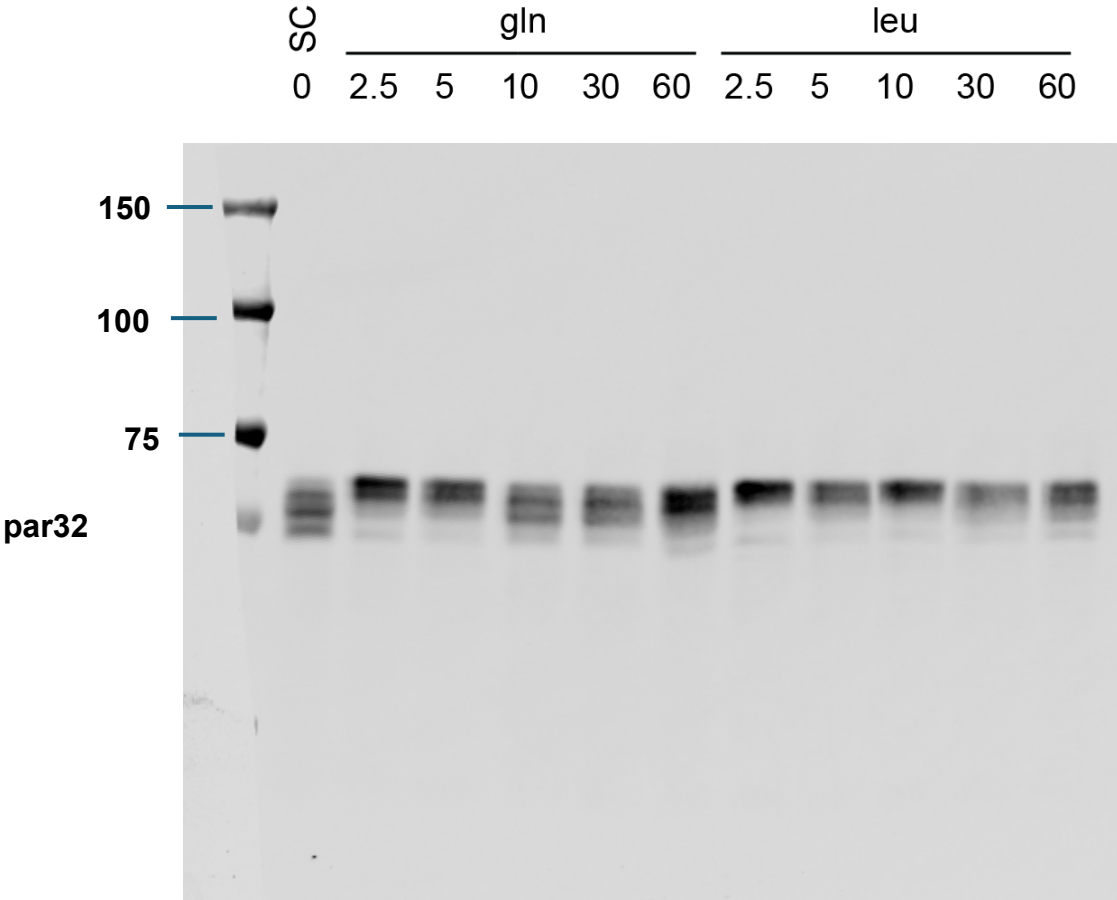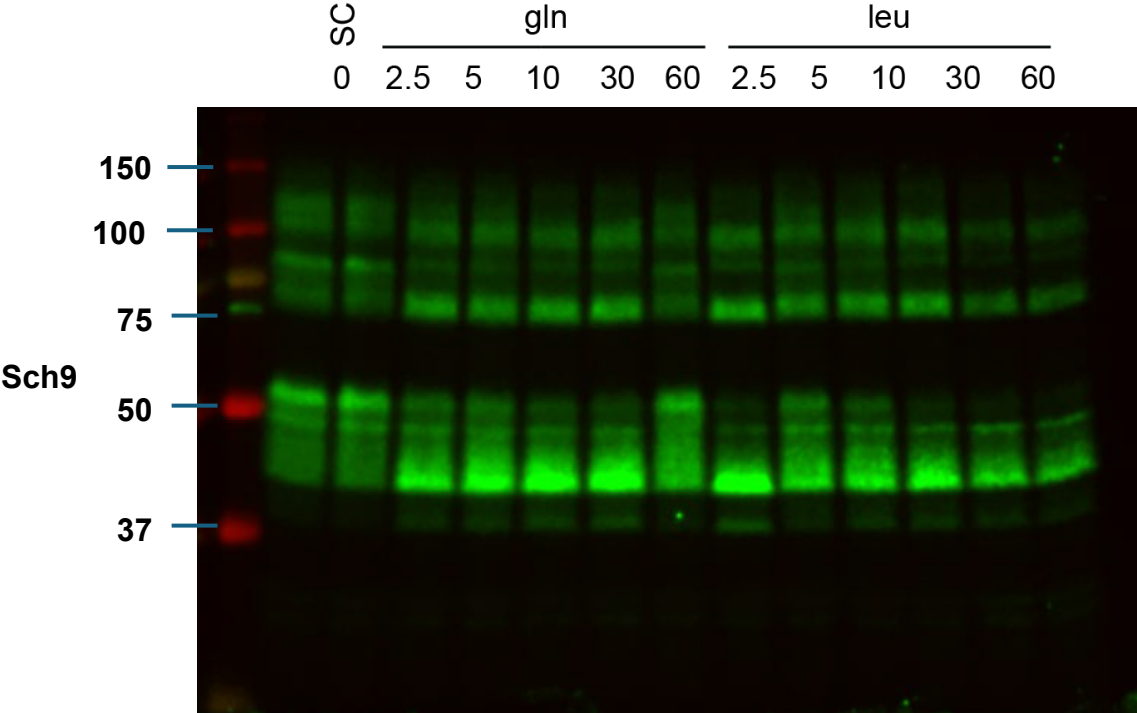

Figure 6C cont

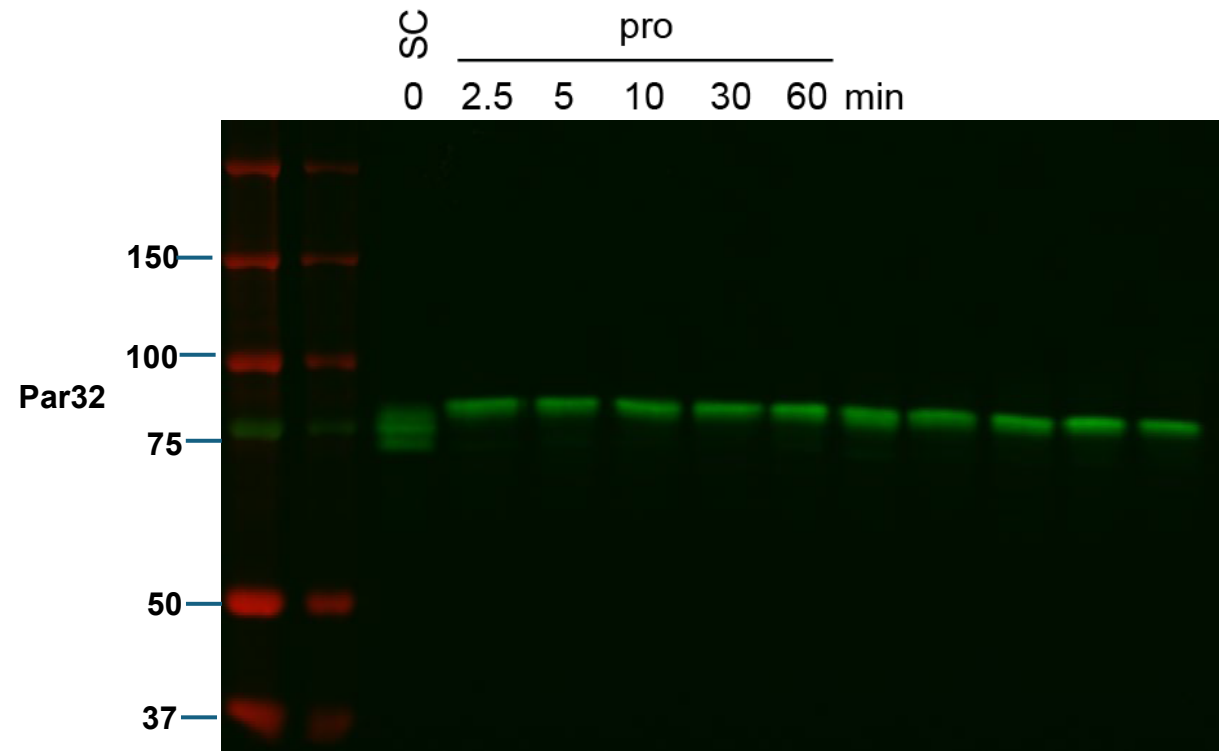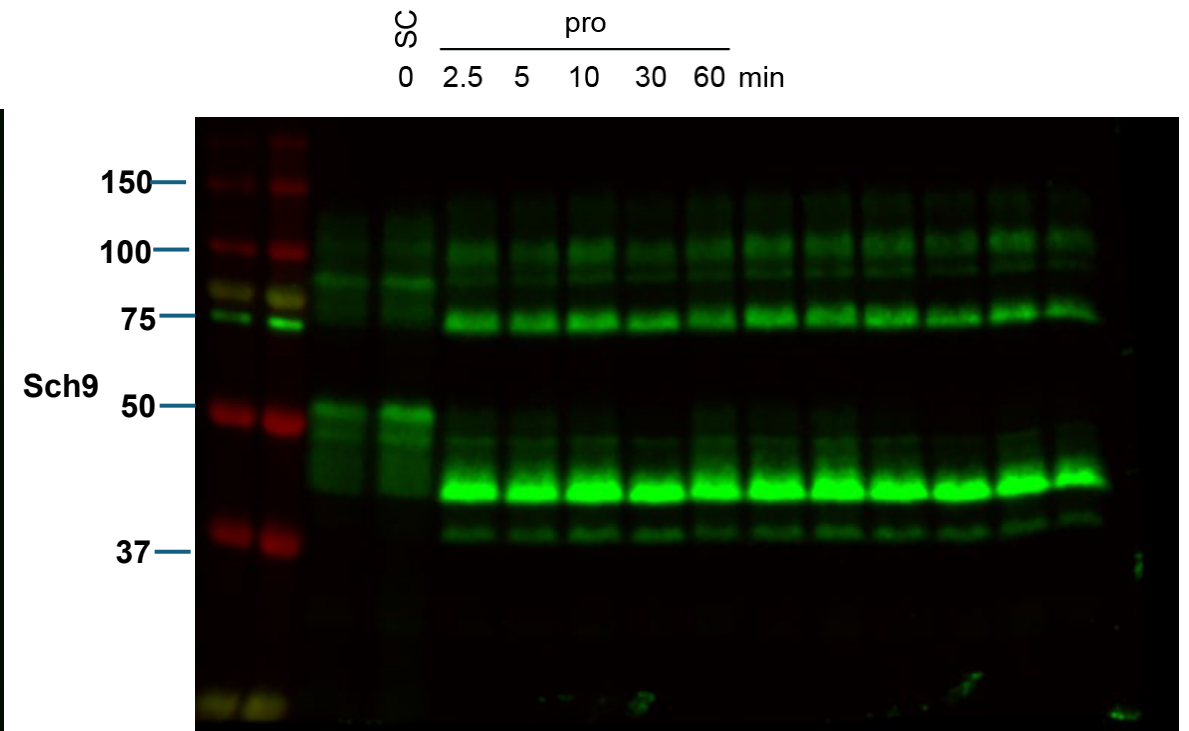

Figure 6D

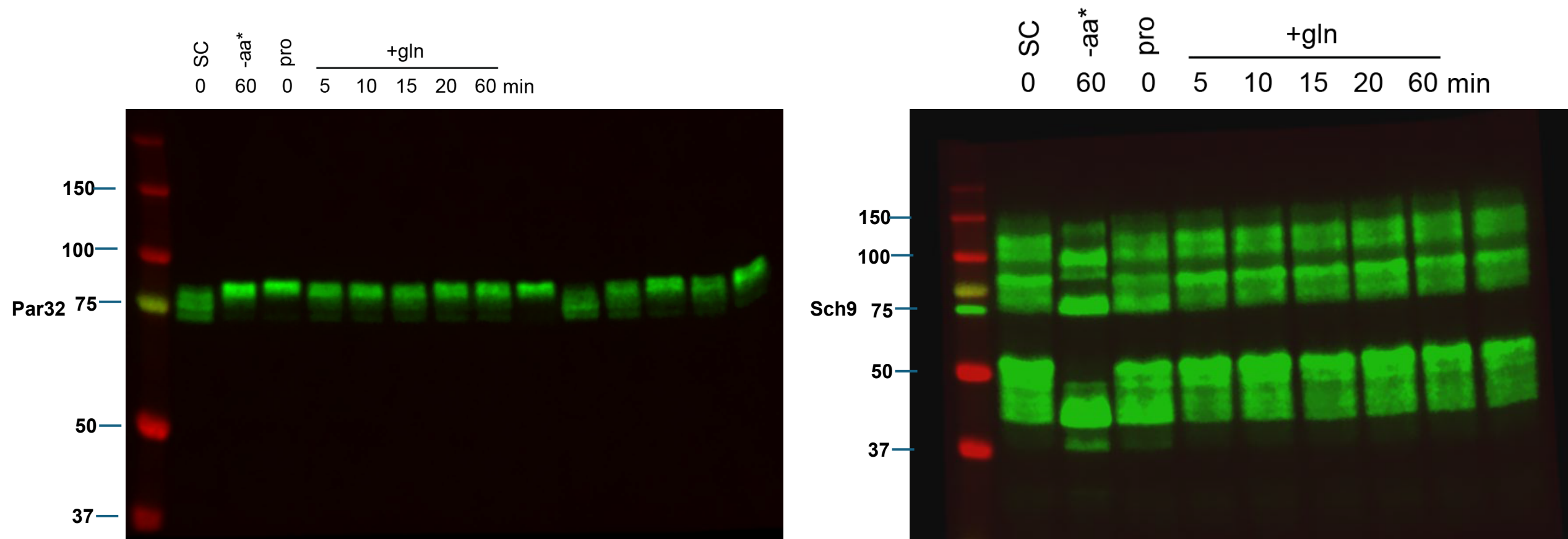

Figure 6E

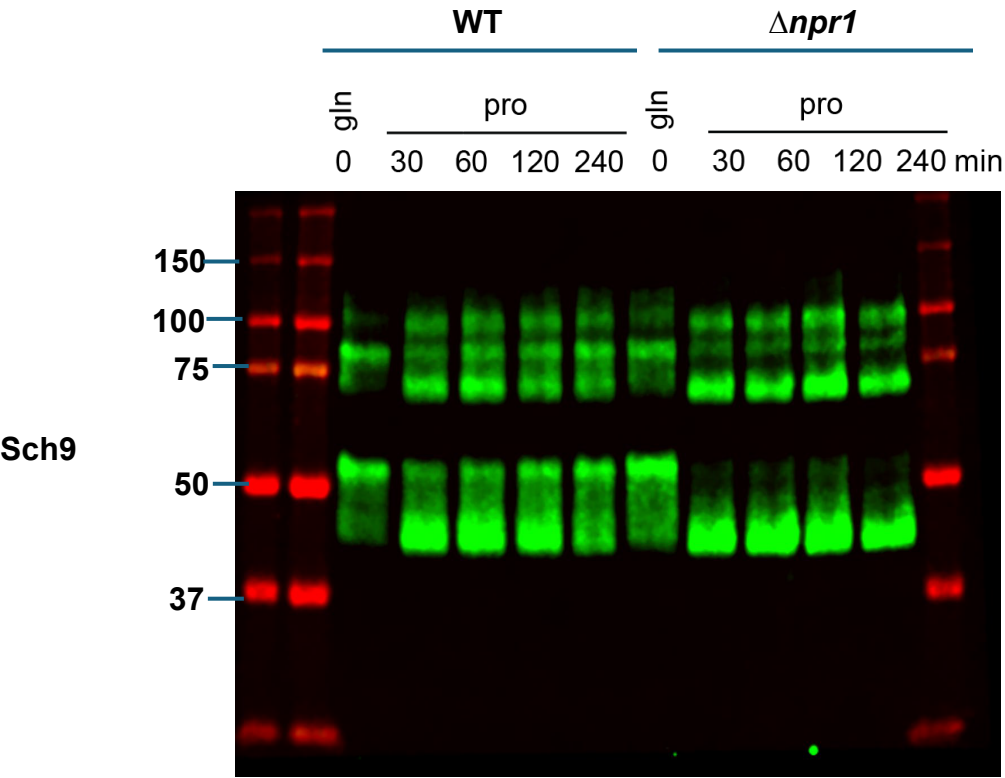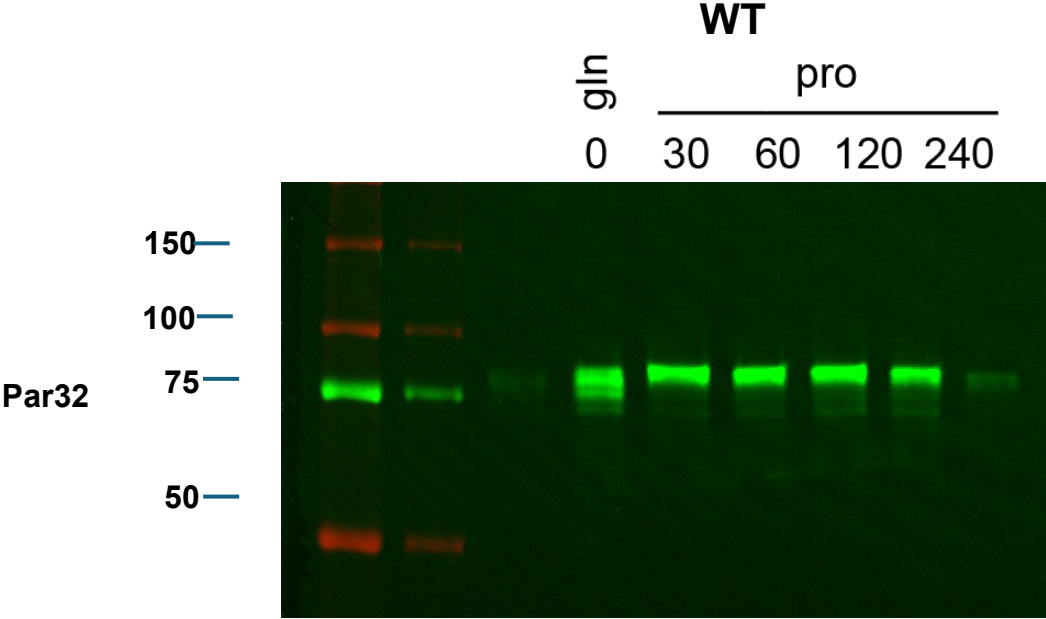

Figure 6E cont

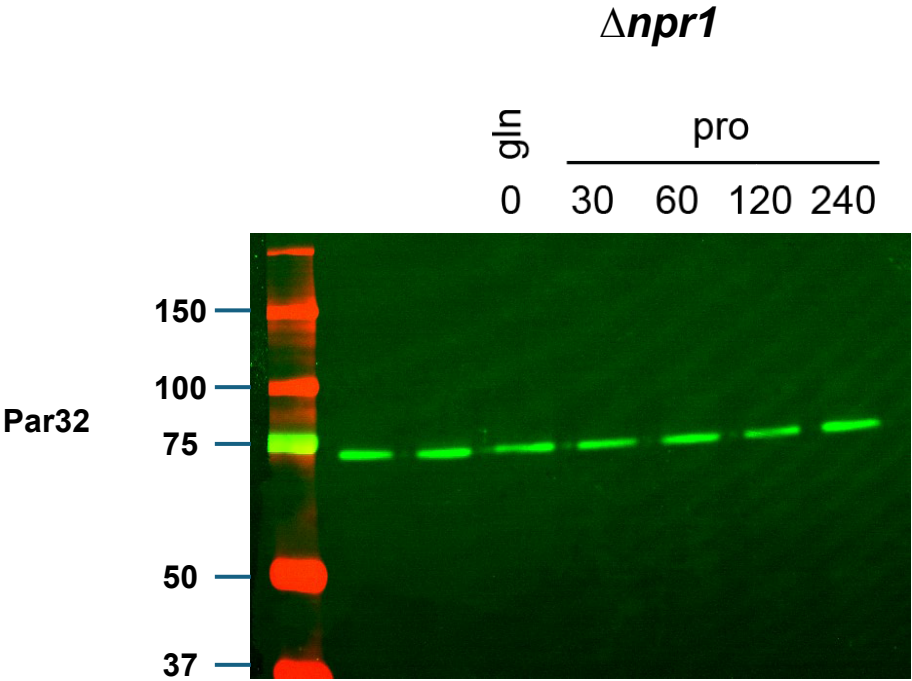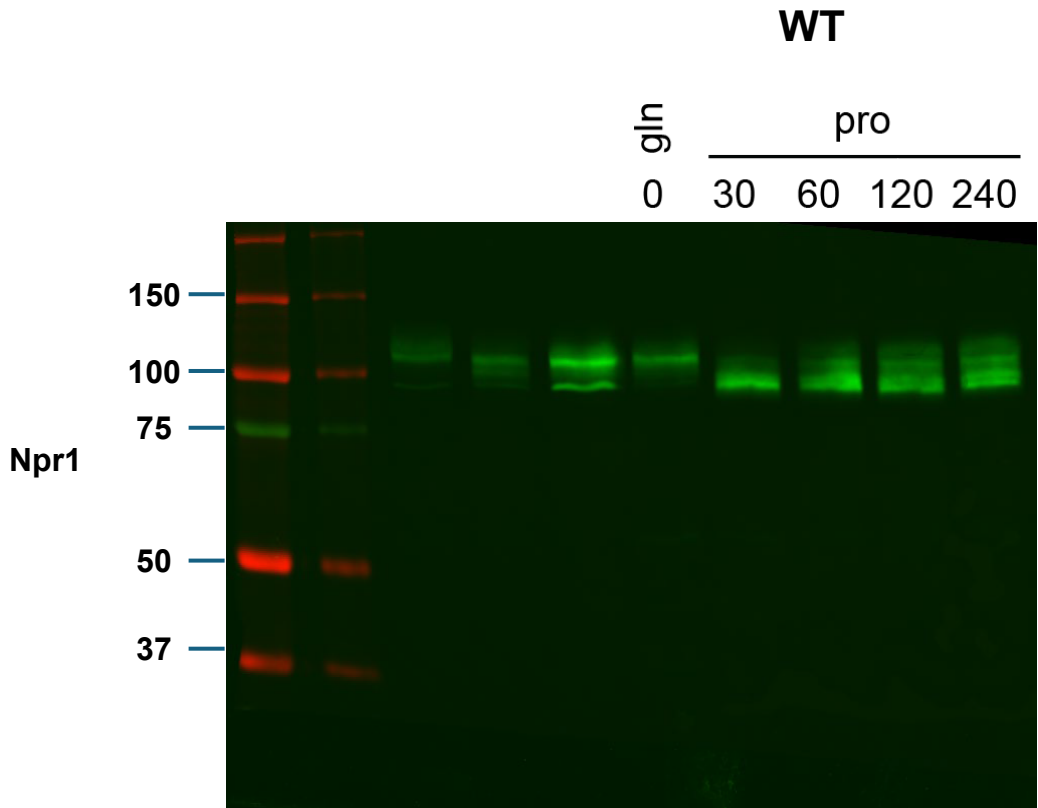

Supplement: Figure 6—source data 1. [file elife-94628-fig6-data1.pdf]

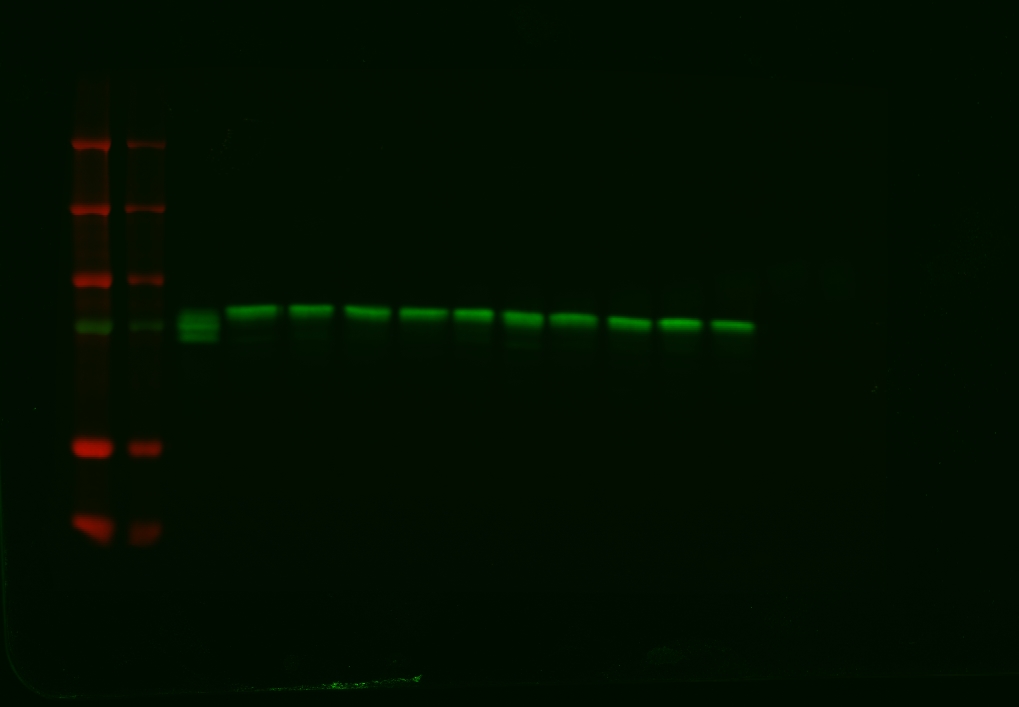

Supplement: Figure 6—source data 2. [file elife-94628-fig6-data2.zip › Figure 6-source data 2/6C_par32_sd_pro_-n.jpg]

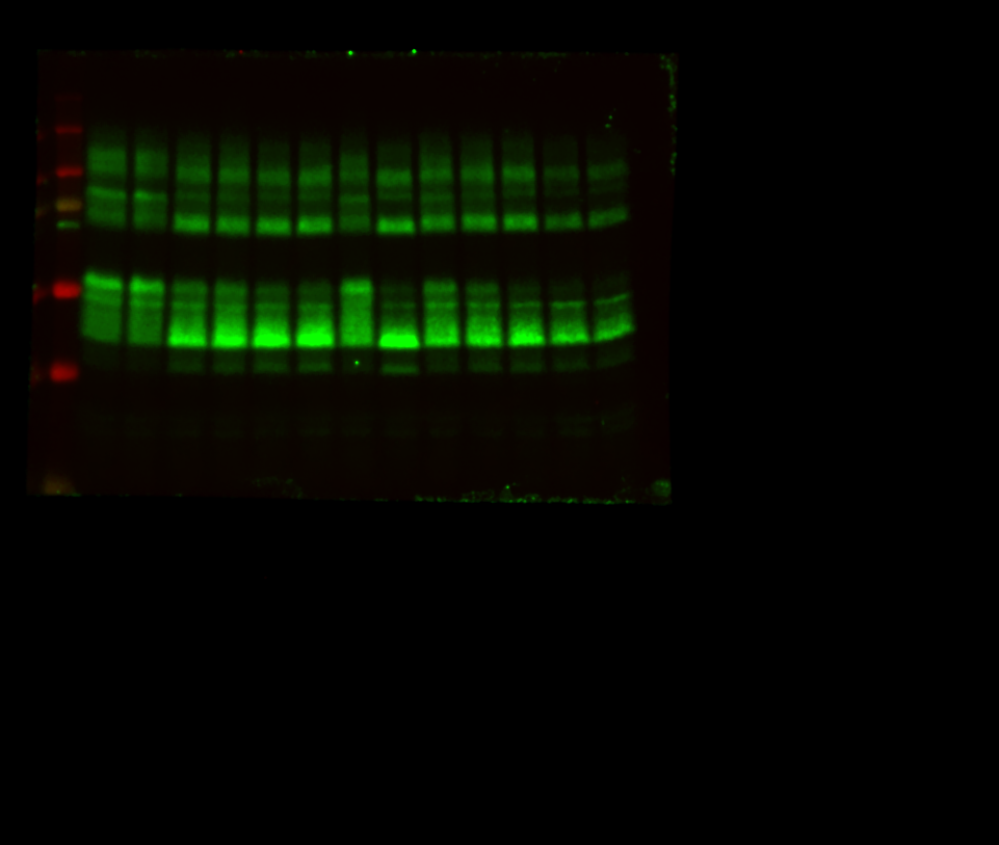

Supplement: Figure 6—source data 2. [file elife-94628-fig6-data2.zip › Figure 6-source data 2/6C_sch9_sd_gln_leu.tif]

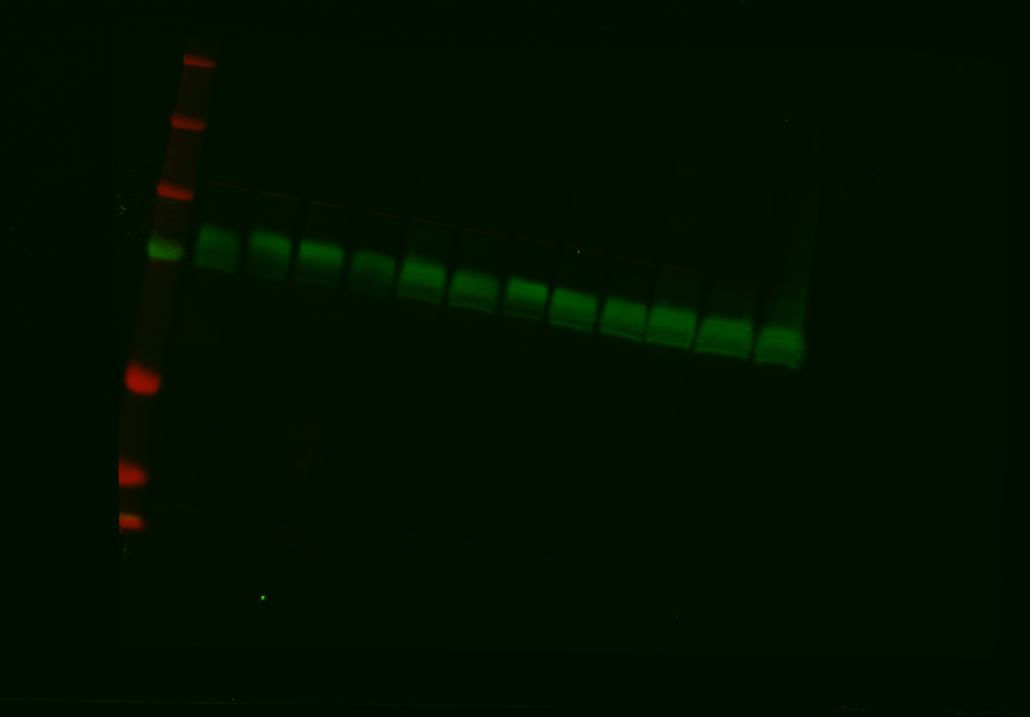

Supplement: Figure 6—source data 2. [file elife-94628-fig6-data2.zip › Figure 6-source data 2/6B_par32_-aas_wt_gtr1ko.tif]

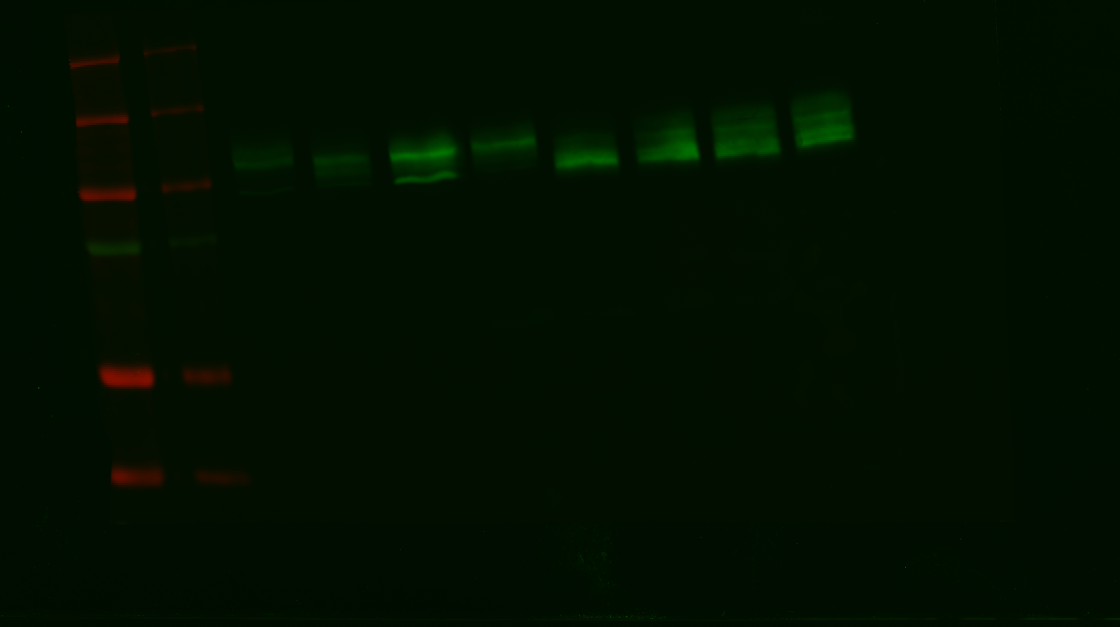

Supplement: Figure 6—source data 2. [file elife-94628-fig6-data2.zip › Figure 6-source data 2/6E_npr1_gln-pro.tif]

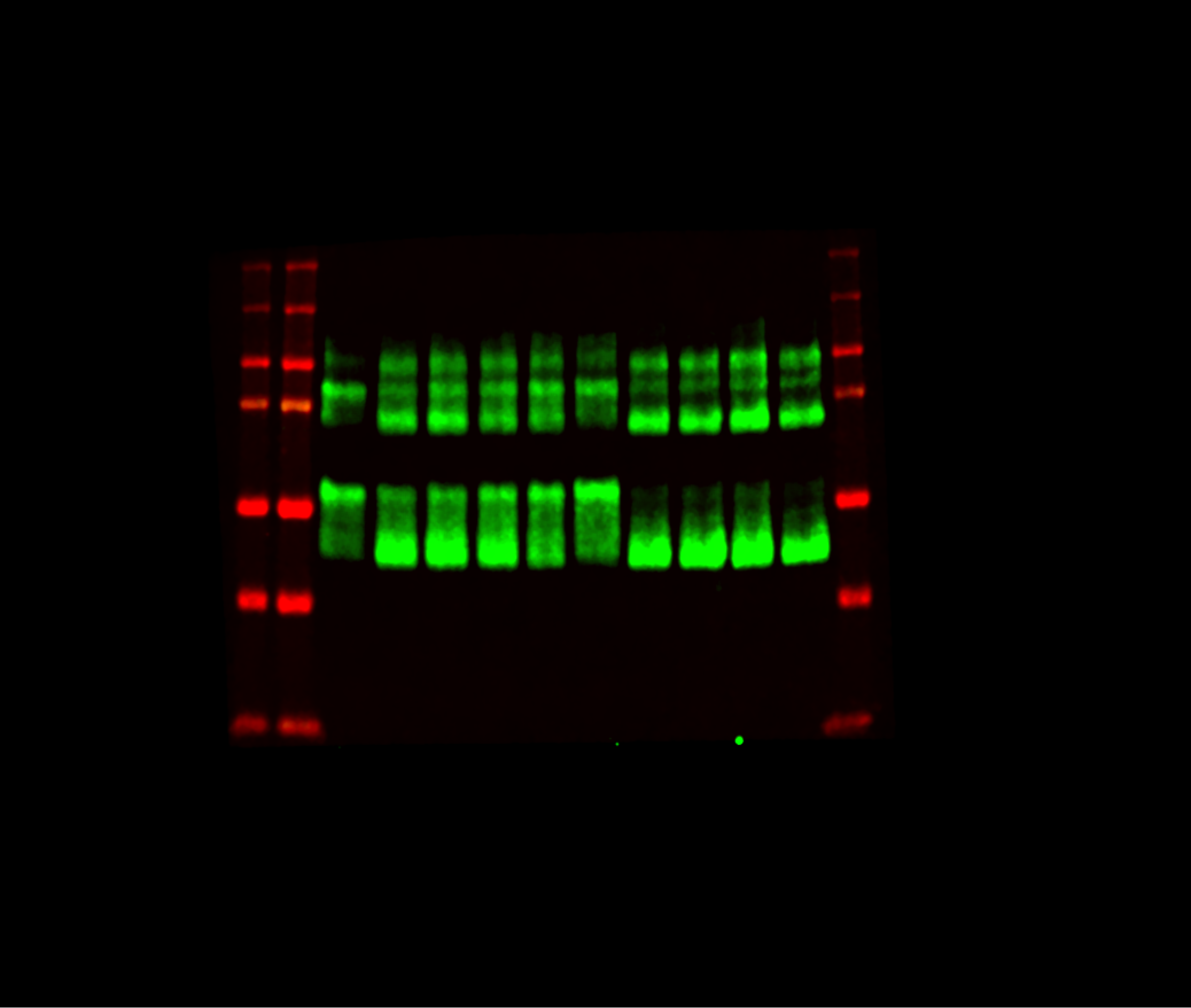

Supplement: Figure 6—source data 2. [file elife-94628-fig6-data2.zip › Figure 6-source data 2/6E_sch9_wt_and_npr1KO_glntopro.tif]

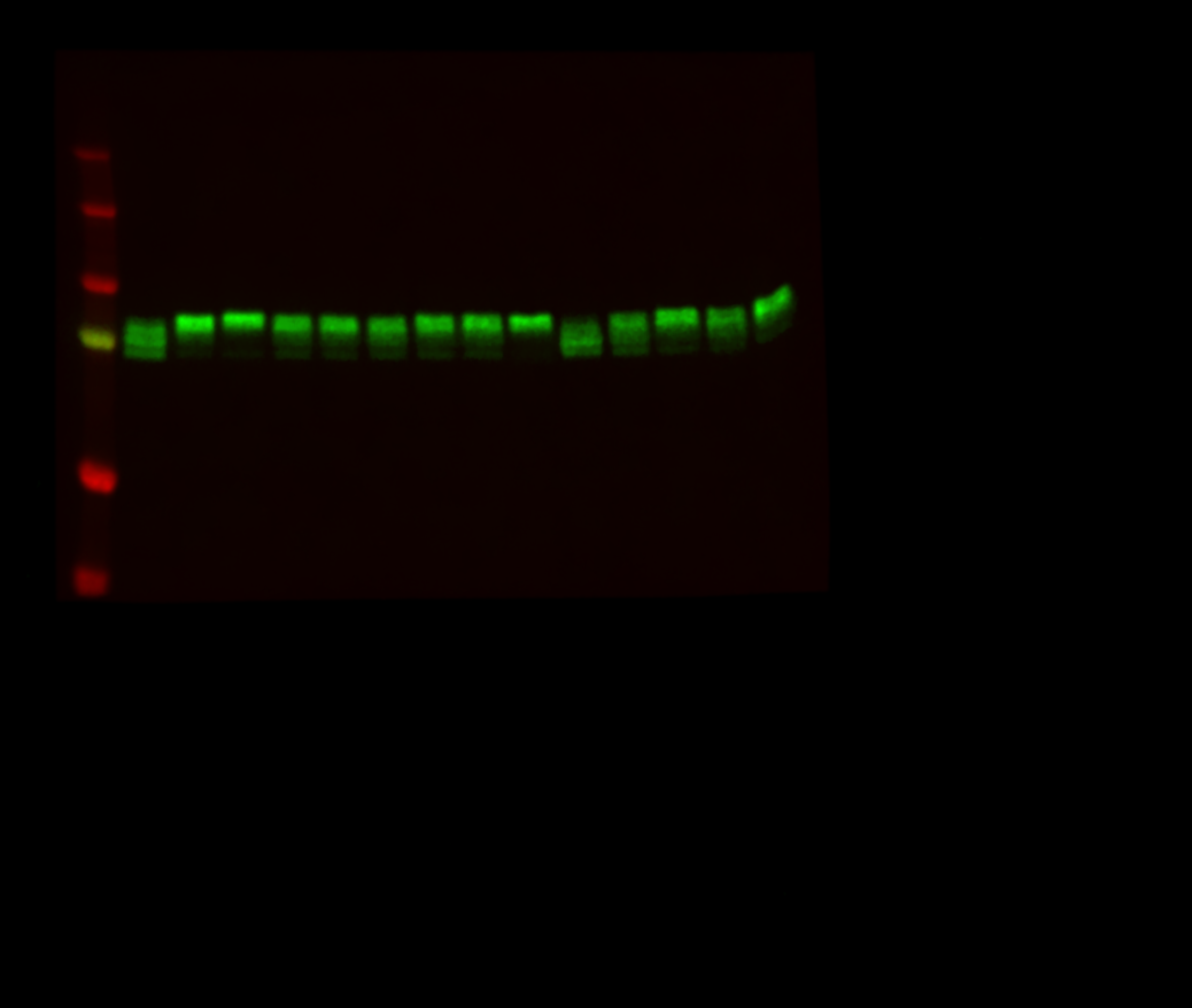

Supplement: Figure 6—source data 2. [file elife-94628-fig6-data2.zip › Figure 6-source data 2/6D_par32.tif]

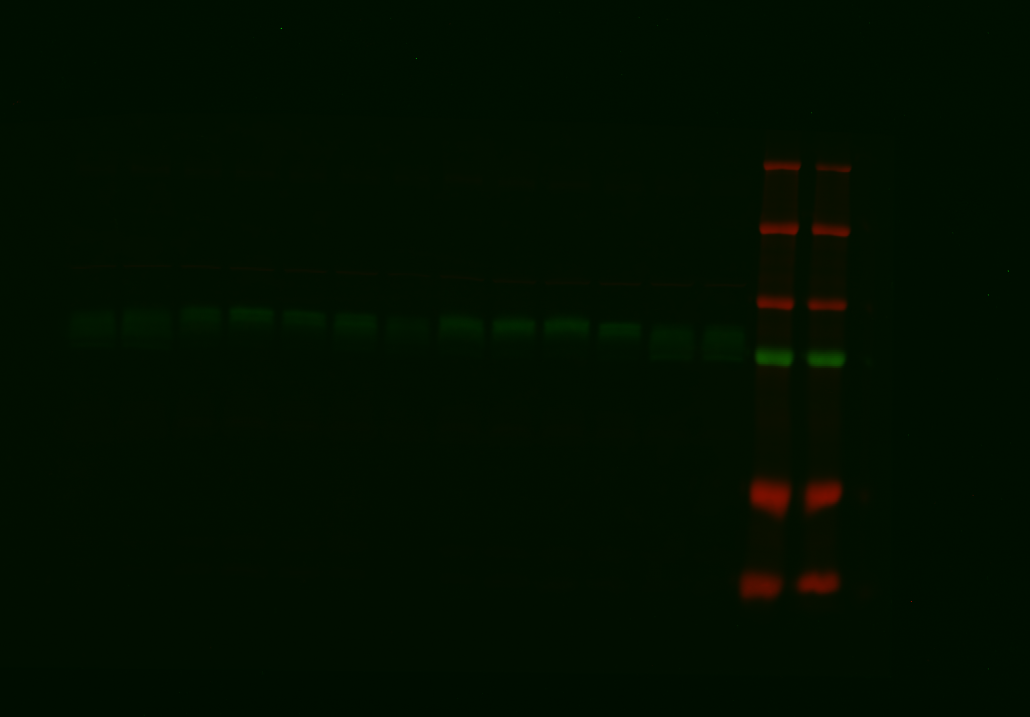

Supplement: Figure 6—source data 2. [file elife-94628-fig6-data2.zip › Figure 6-source data 2/6B_par32_-aas_pib2ko.tif]

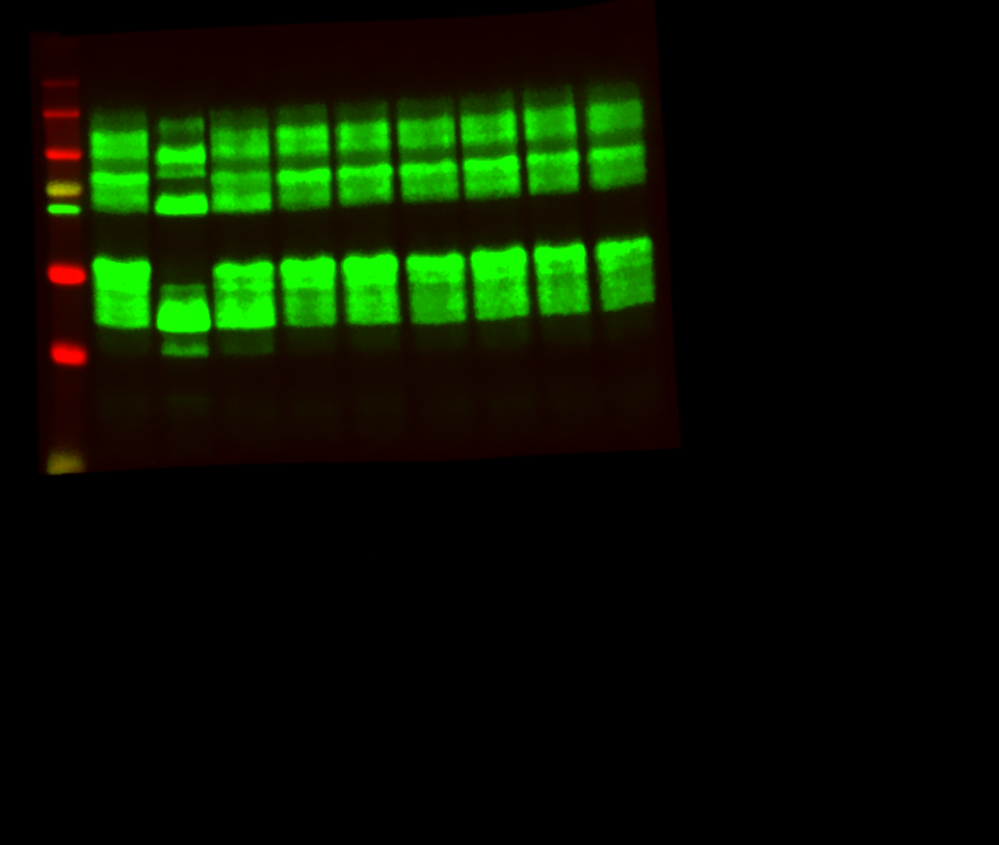

Supplement: Figure 6—source data 2. [file elife-94628-fig6-data2.zip › Figure 6-source data 2/6D_sch9_wt.tif]

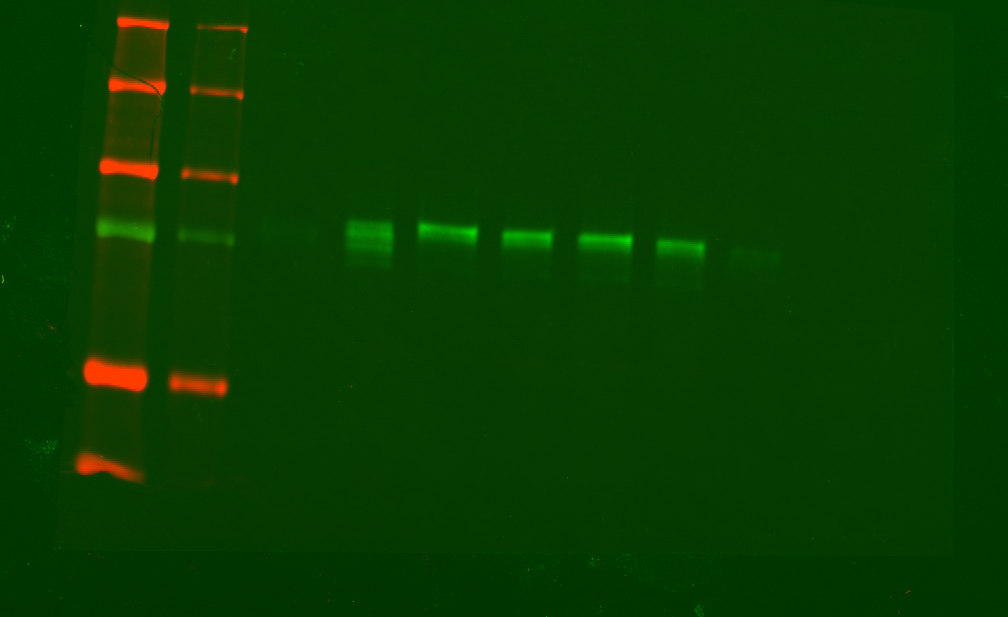

Supplement: Figure 6—source data 2. [file elife-94628-fig6-data2.zip › Figure 6-source data 2/6E_par32_pro-downshift.tif]

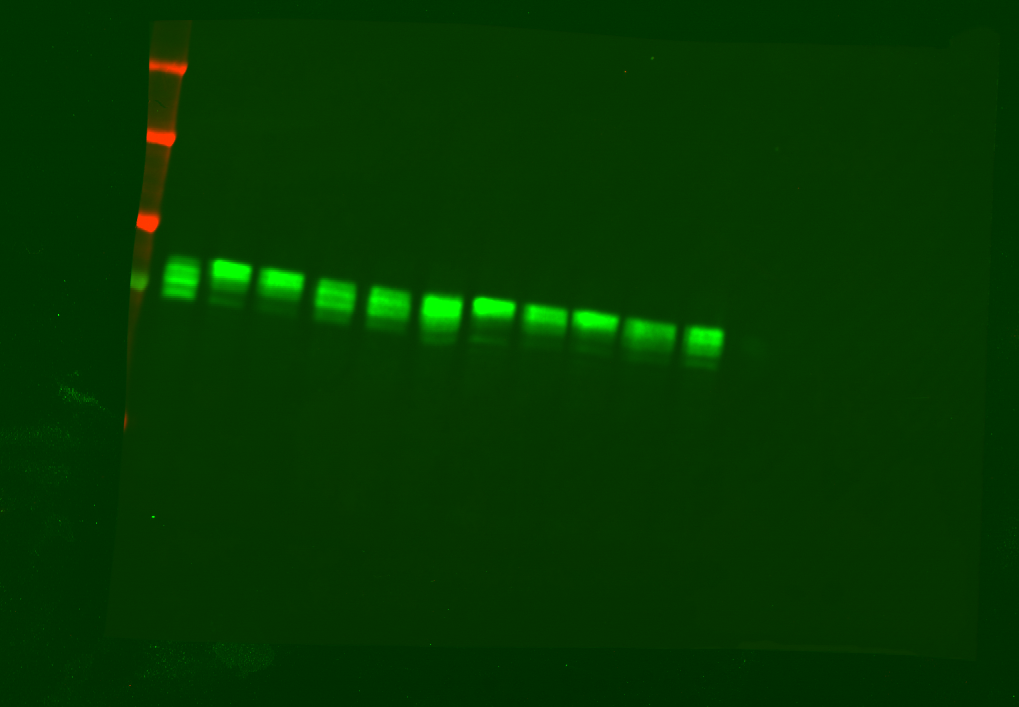

Supplement: Figure 6—source data 2. [file elife-94628-fig6-data2.zip › Figure 6-source data 2/6C_par32_sd_gln_leu.tif]

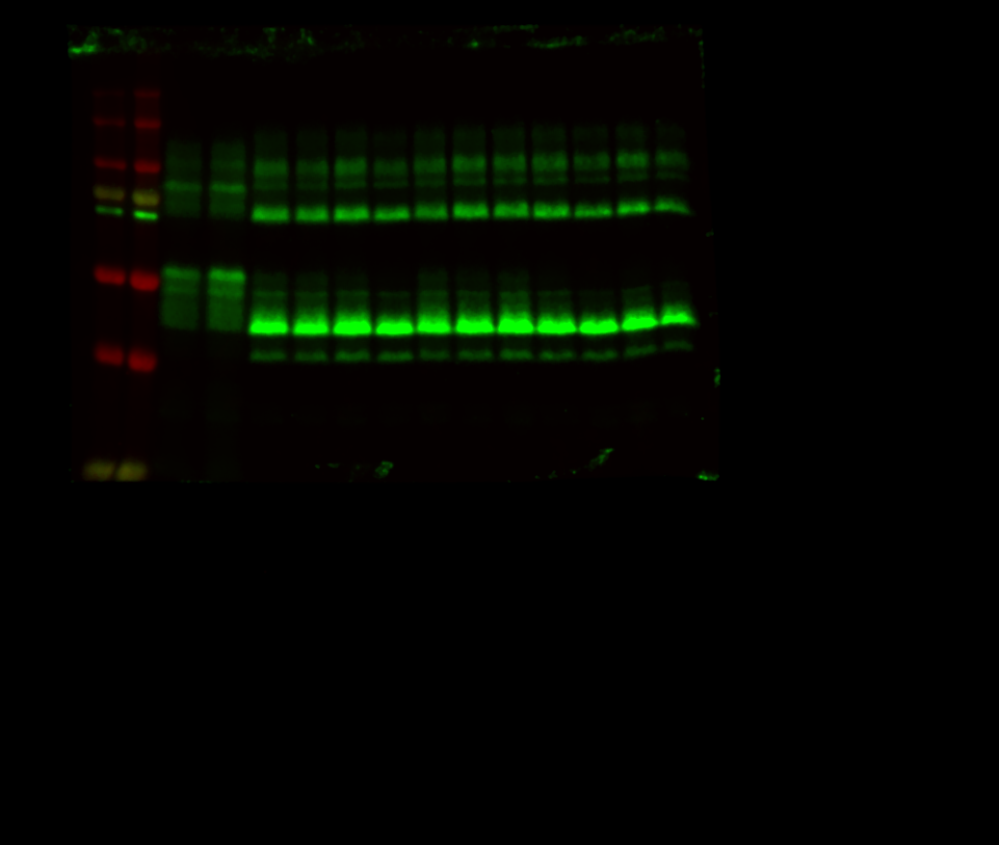

Supplement: Figure 6—source data 2. [file elife-94628-fig6-data2.zip › Figure 6-source data 2/6C_sch9_sd-pro_-n.tif]

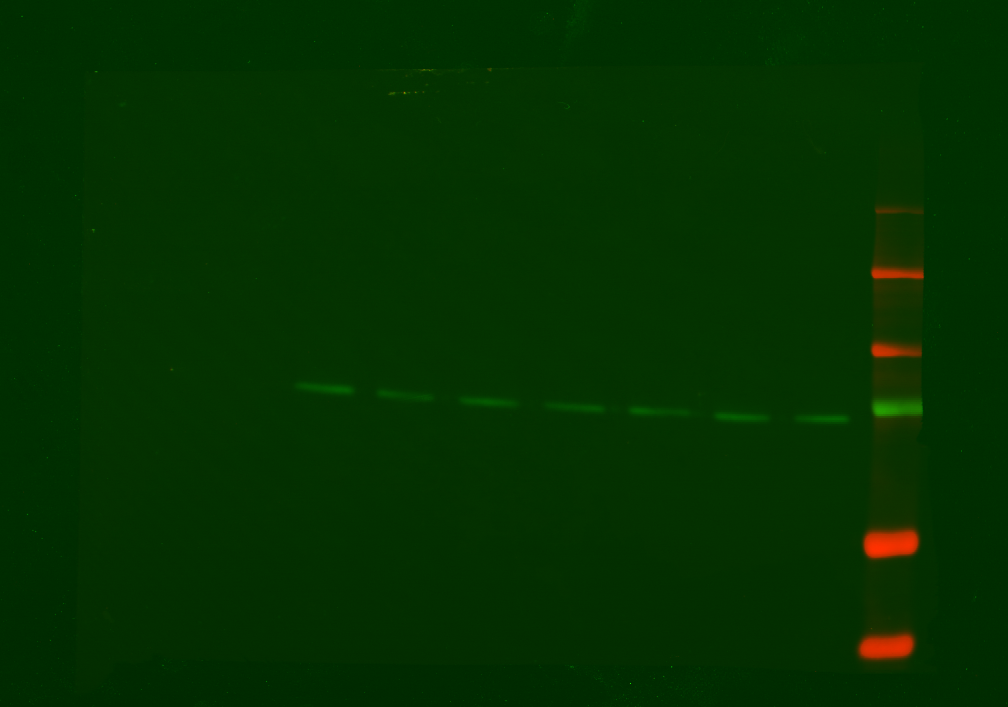

Supplement: Figure 6—source data 2. [file elife-94628-fig6-data2.zip › Figure 6-source data 2/6E_npr1ko_par32.tif]

Figure 8A

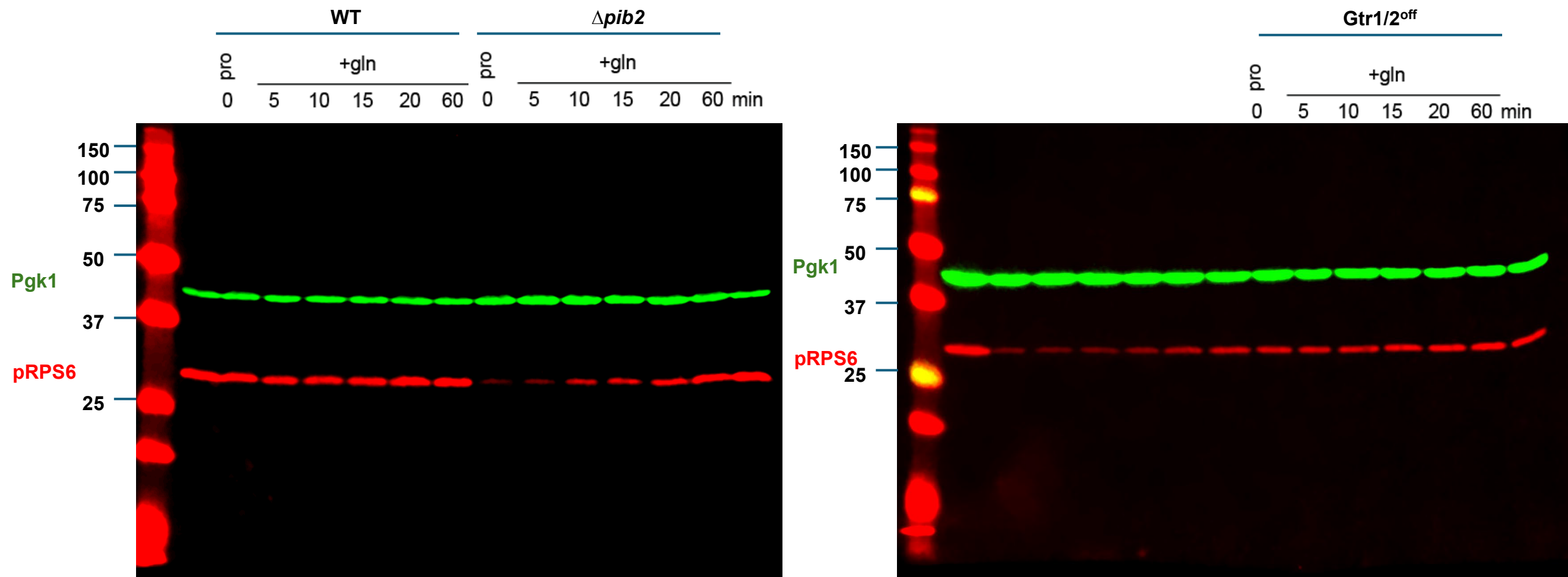

Figure 8B

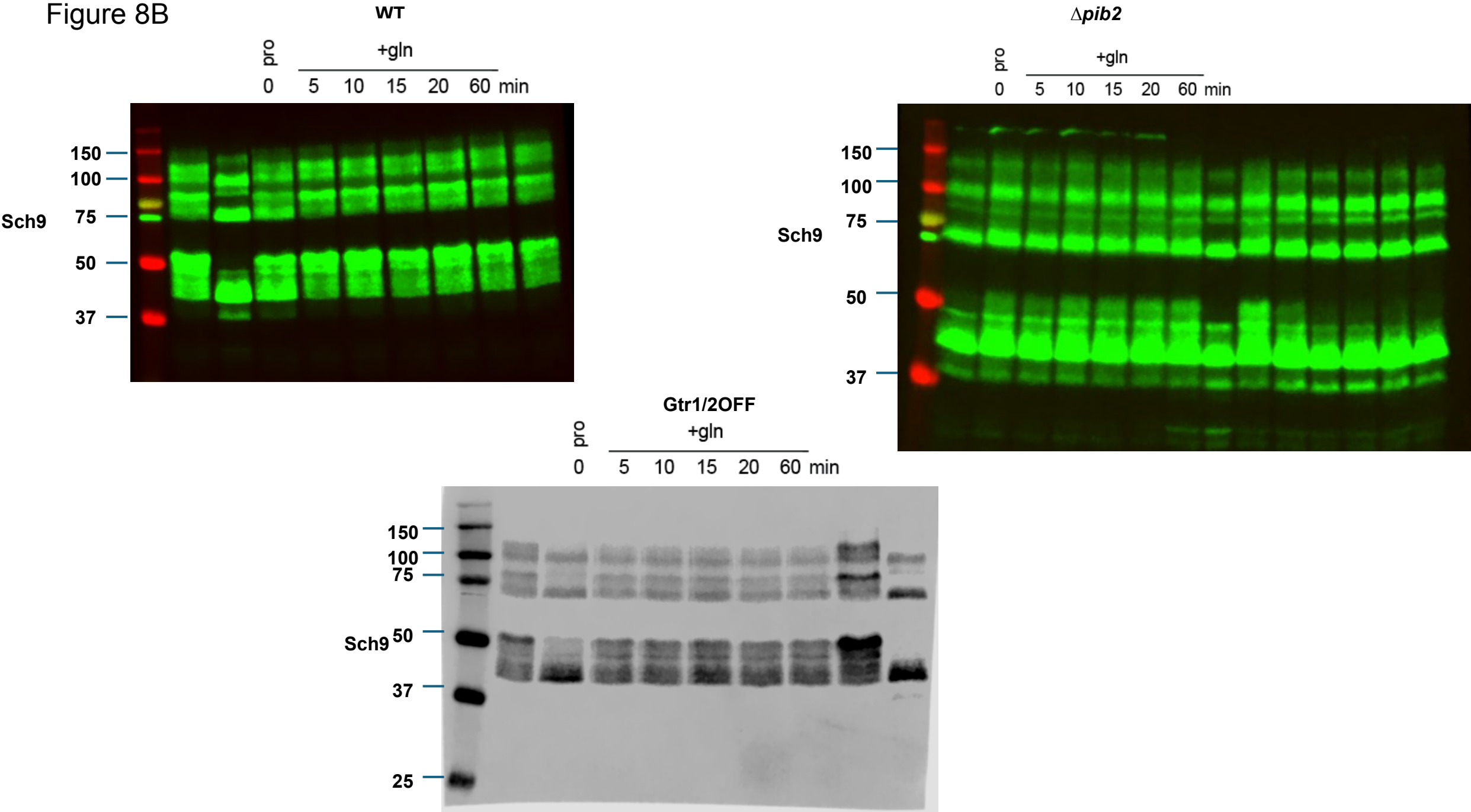

Figure 8C

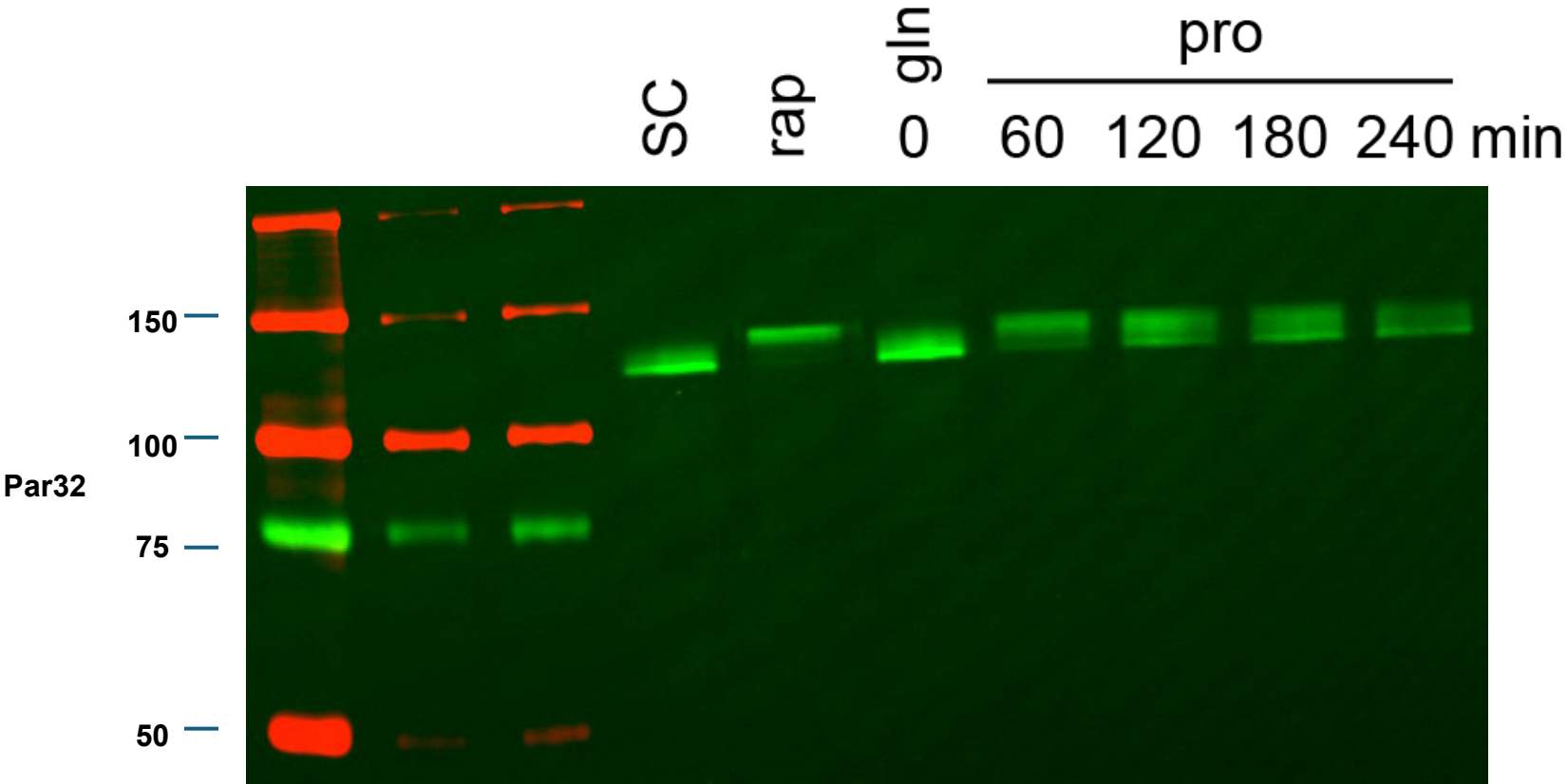

Supplement: Figure 8—source data 1. [file elife-94628-fig8-data1.pdf]

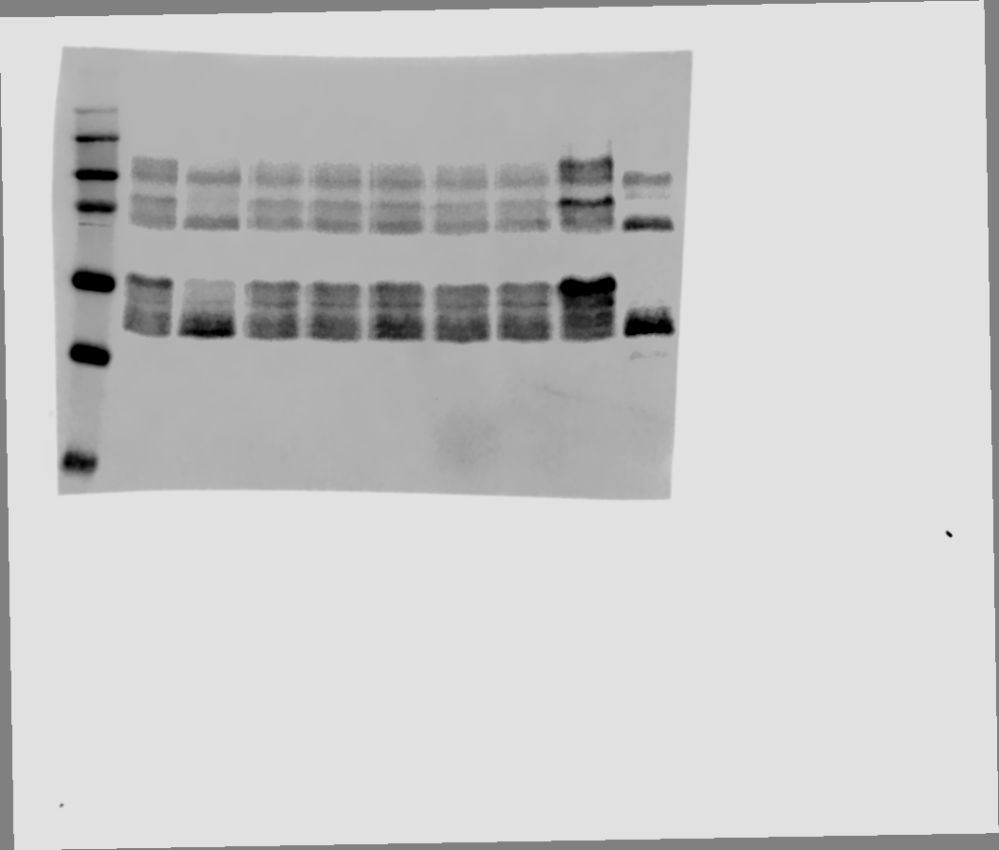

Supplement: Figure 8—source data 2. [file elife-94628-fig8-data2.zip › Figure 8-source data 2/8B_sch9_gtr1-2_off.tif]

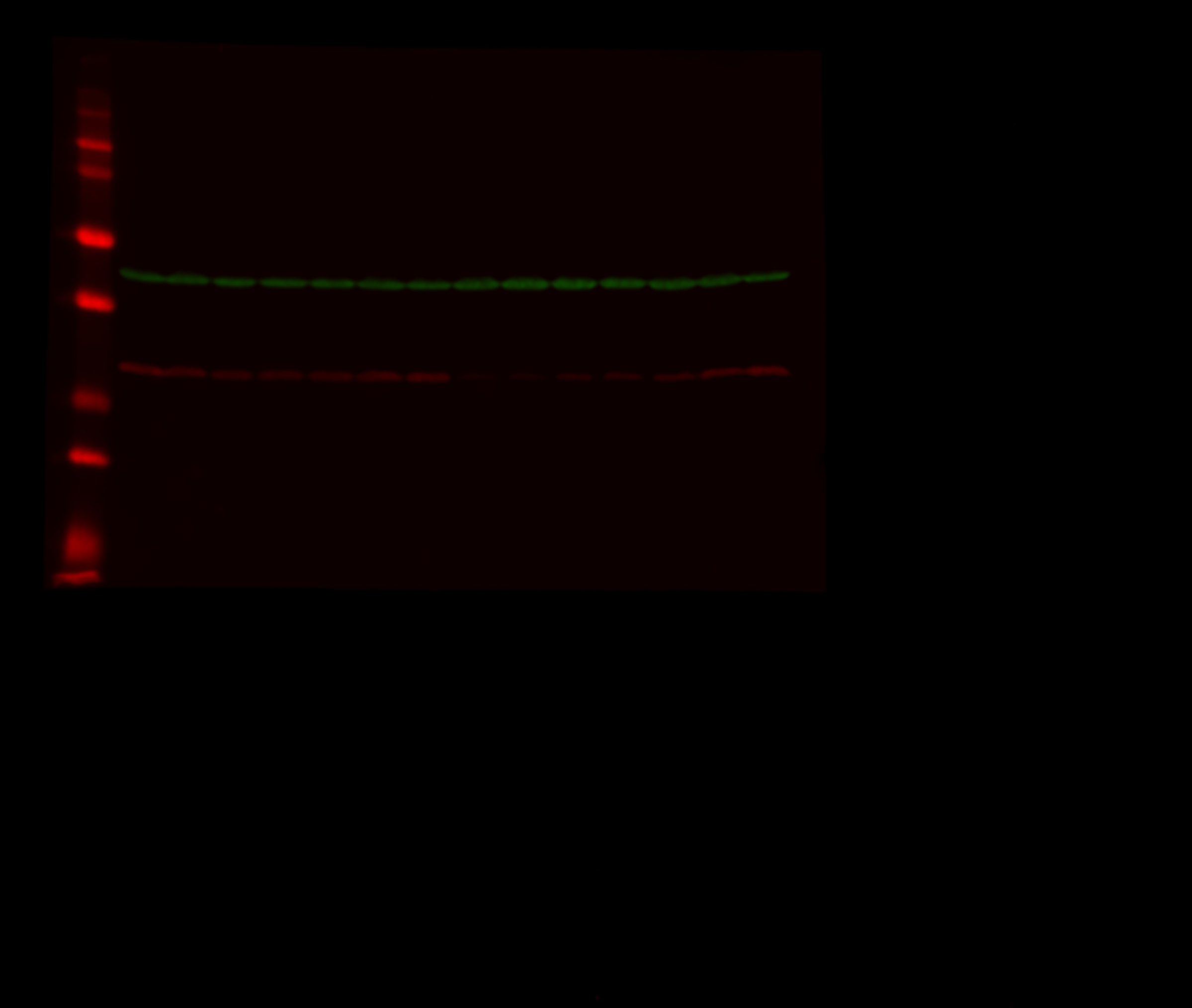

Supplement: Figure 8—source data 2. [file elife-94628-fig8-data2.zip › Figure 8-source data 2/8A_rps6_pro-gln_upshift_wt_pib2.tif]

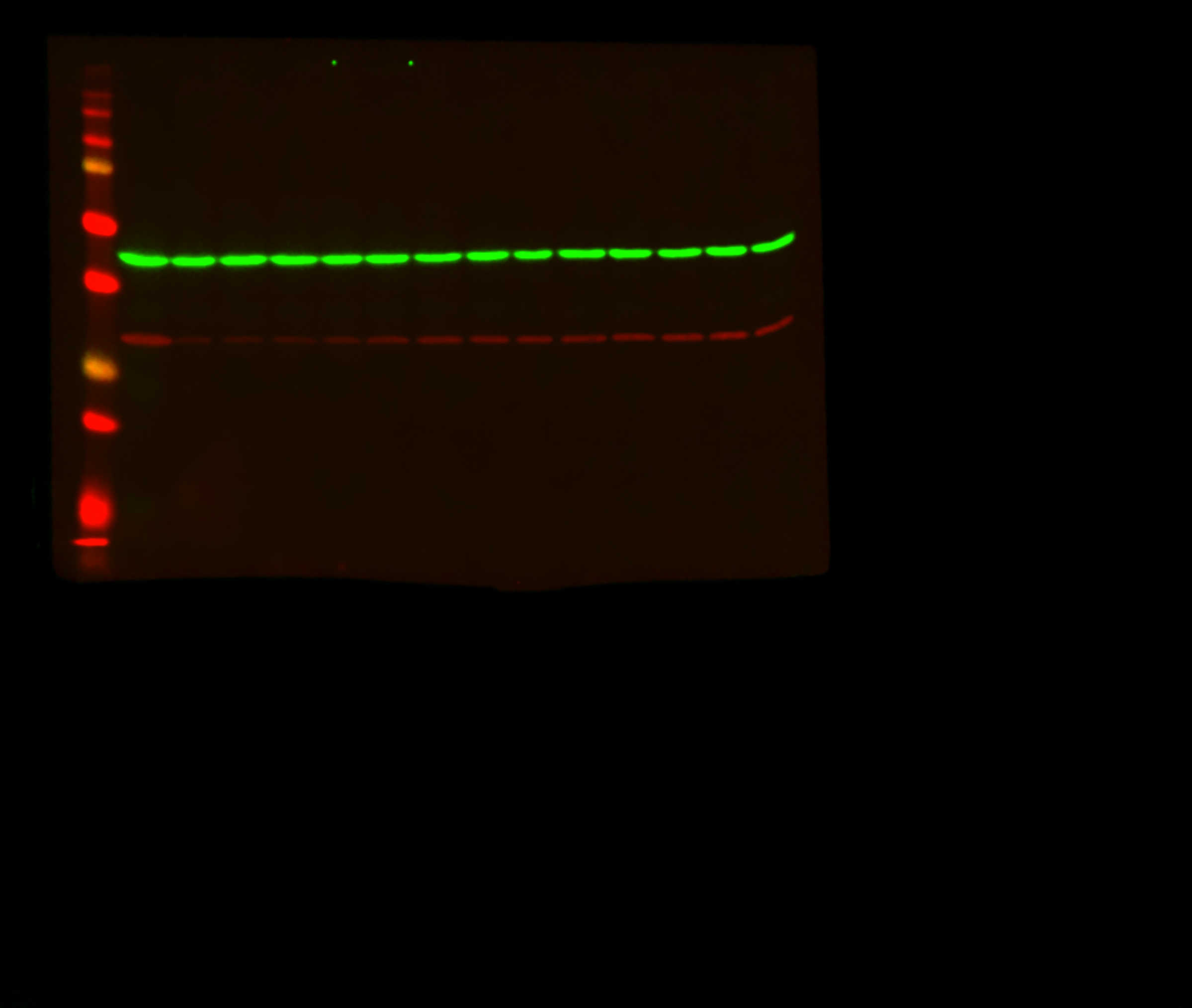

Supplement: Figure 8—source data 2. [file elife-94628-fig8-data2.zip › Figure 8-source data 2/8A_rps6_gtroff.tif]

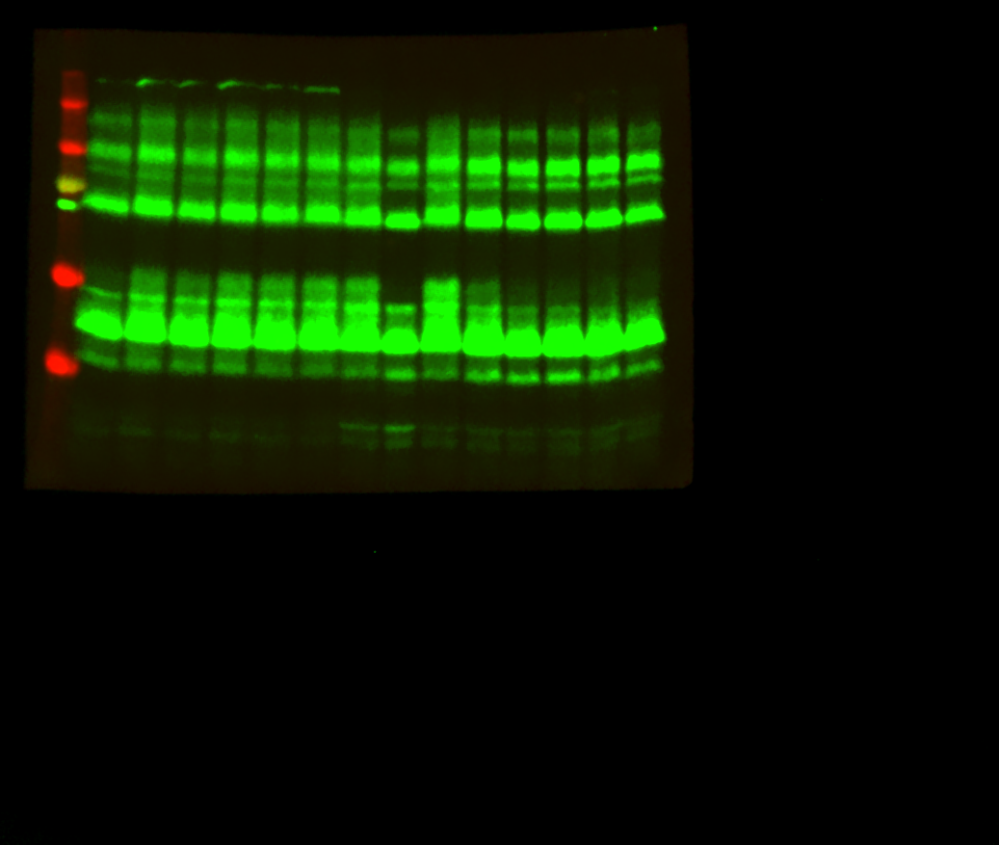

Supplement: Figure 8—source data 2. [file elife-94628-fig8-data2.zip › Figure 8-source data 2/8B_sch9_pib2KO_left-6-samples.tif]

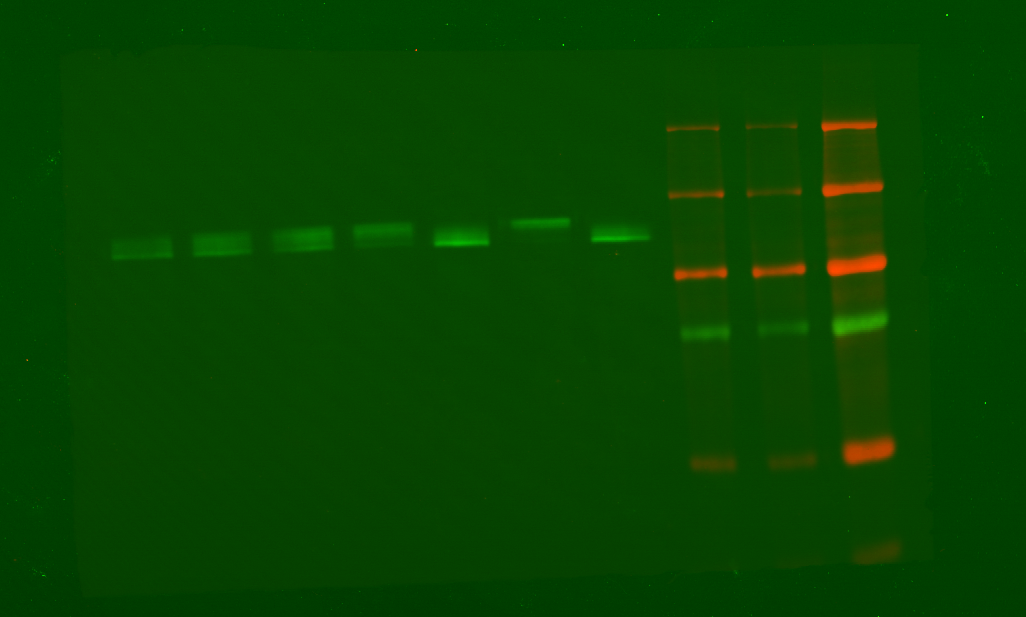

Supplement: Figure 8—source data 2. [file elife-94628-fig8-data2.zip › Figure 8-source data 2/8C_gtr1ON_par32_gln-pro.tif]
