# Supplementary material for: The molecular logic of Gtr1/2- and Pib2-dependent TORC1 regulation in budding yeast
Source: eLife. 2025 Jul 7;13:RP94628. doi: 10.7554/eLife.94628 (PMC12234008; doi:10.7554/eLife.94628)

Figure 5B

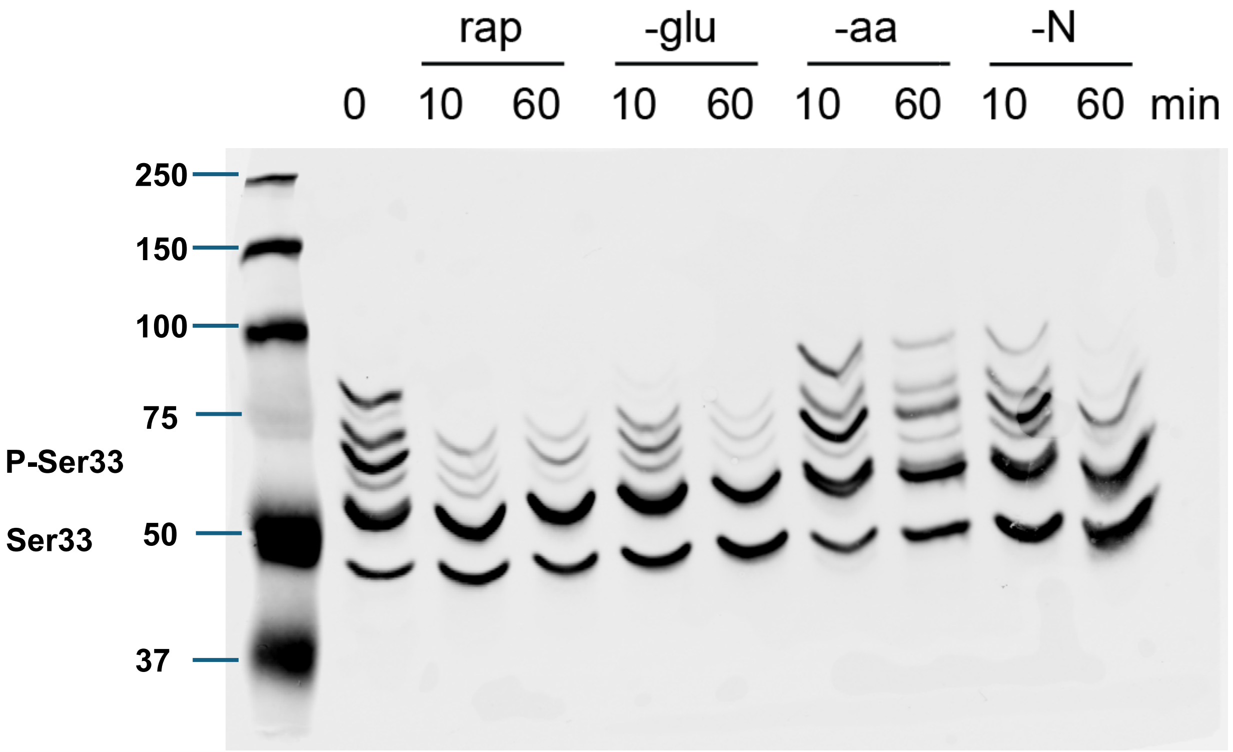

rap
-glu
-aa
-N
0 10 60 10 60 10 60 10 60 min
250
150
100
75
P-Ser33
Ser33
50
37

Figure 5C

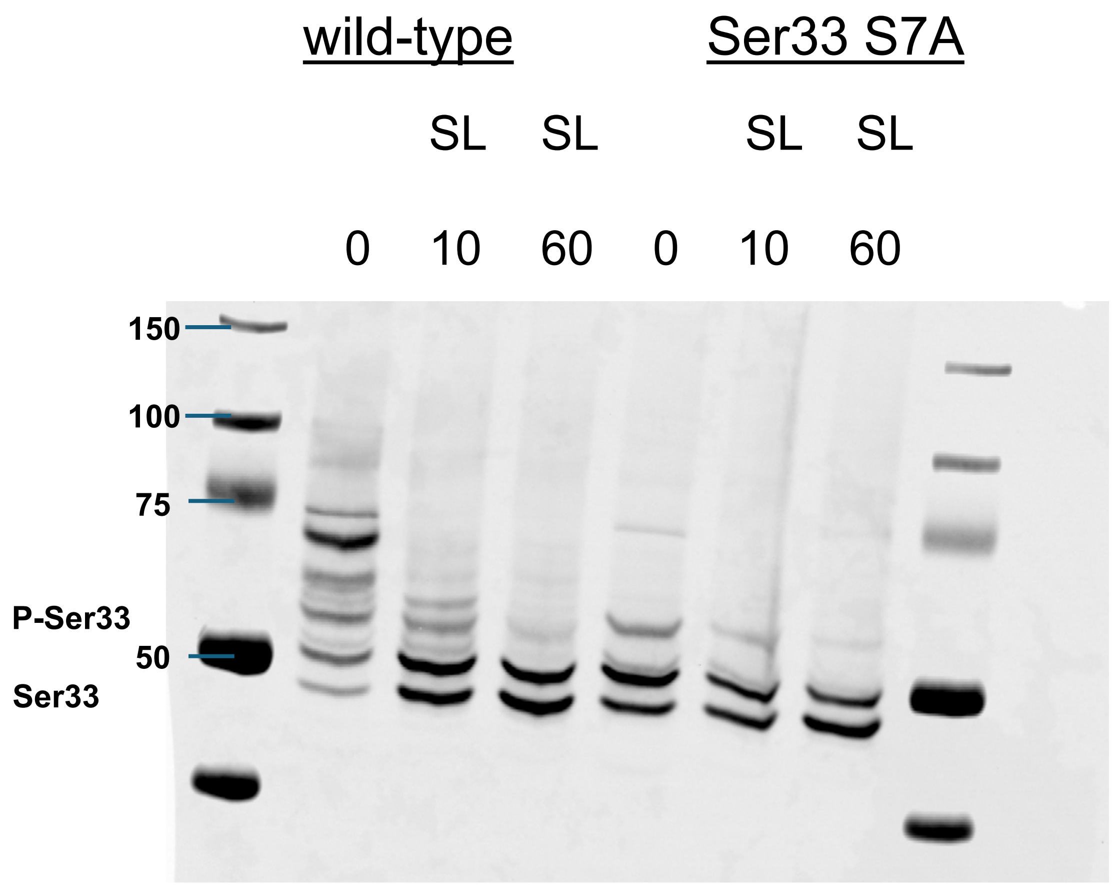
wild-type
Ser33 S7A
SL SL SL SL
0 10 60 0 10 60
150
100
75
P-Ser33
50
Ser33

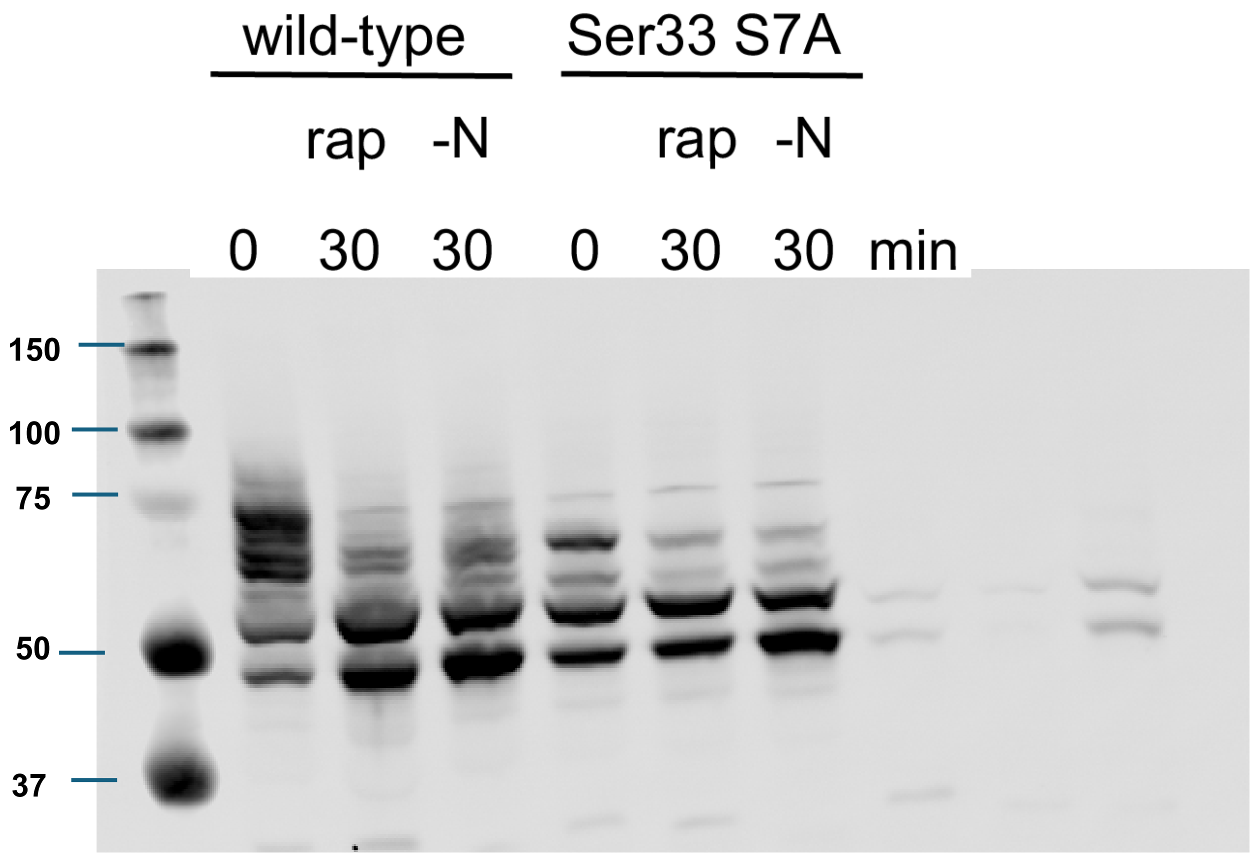
wild-type
Ser33 S7A
rap -N rap -N
0 30 30 0 30 30 min
150
100
75
50
37

Figure 5D

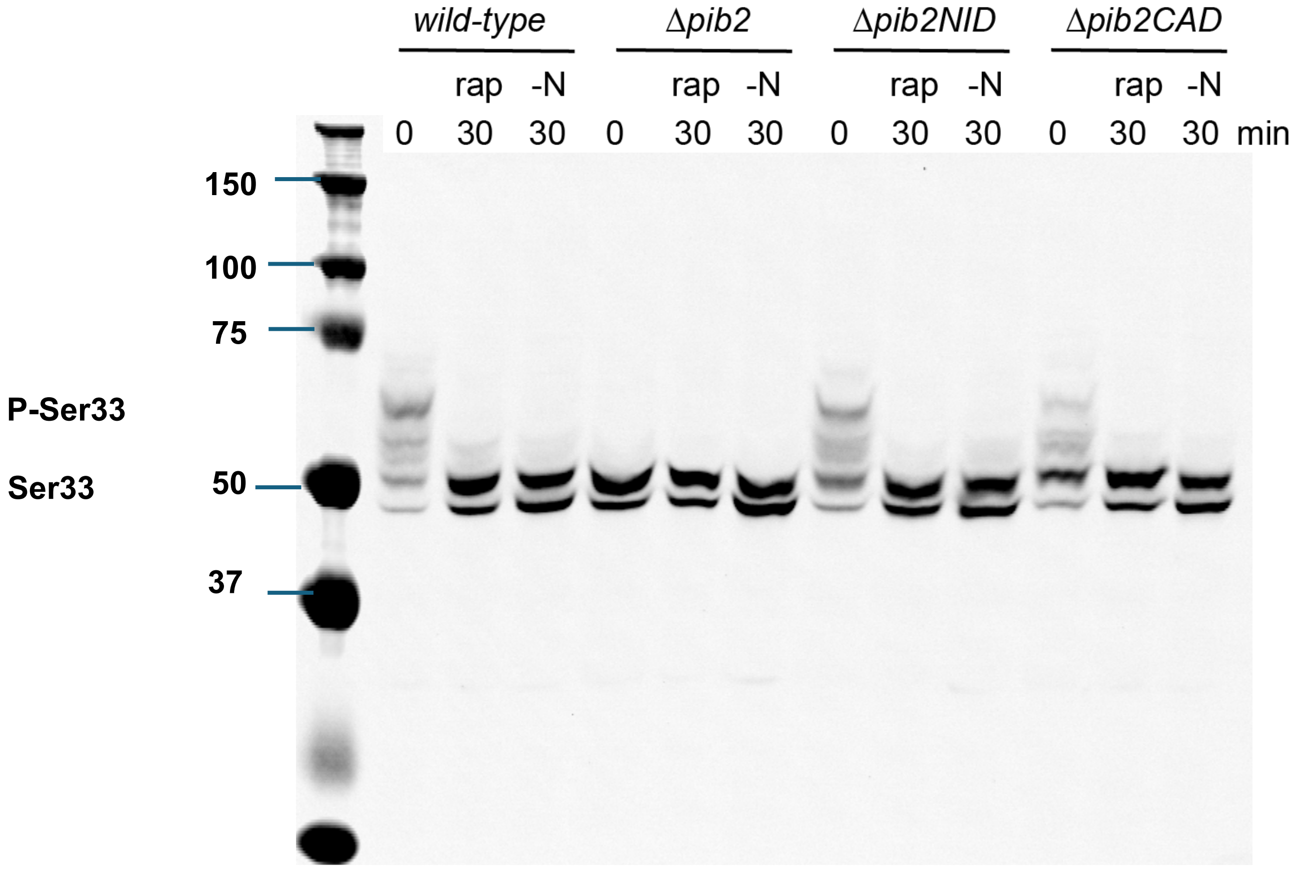
wild-type
Δpib2
Δpib2NID
Δpib2CAD
rap -N rap -N rap -N rap -N
0 30 30 0 30 30 0 30 30 0 30 30 min
150
100
75
P-Ser33
50
Ser33
37

Figure 5E

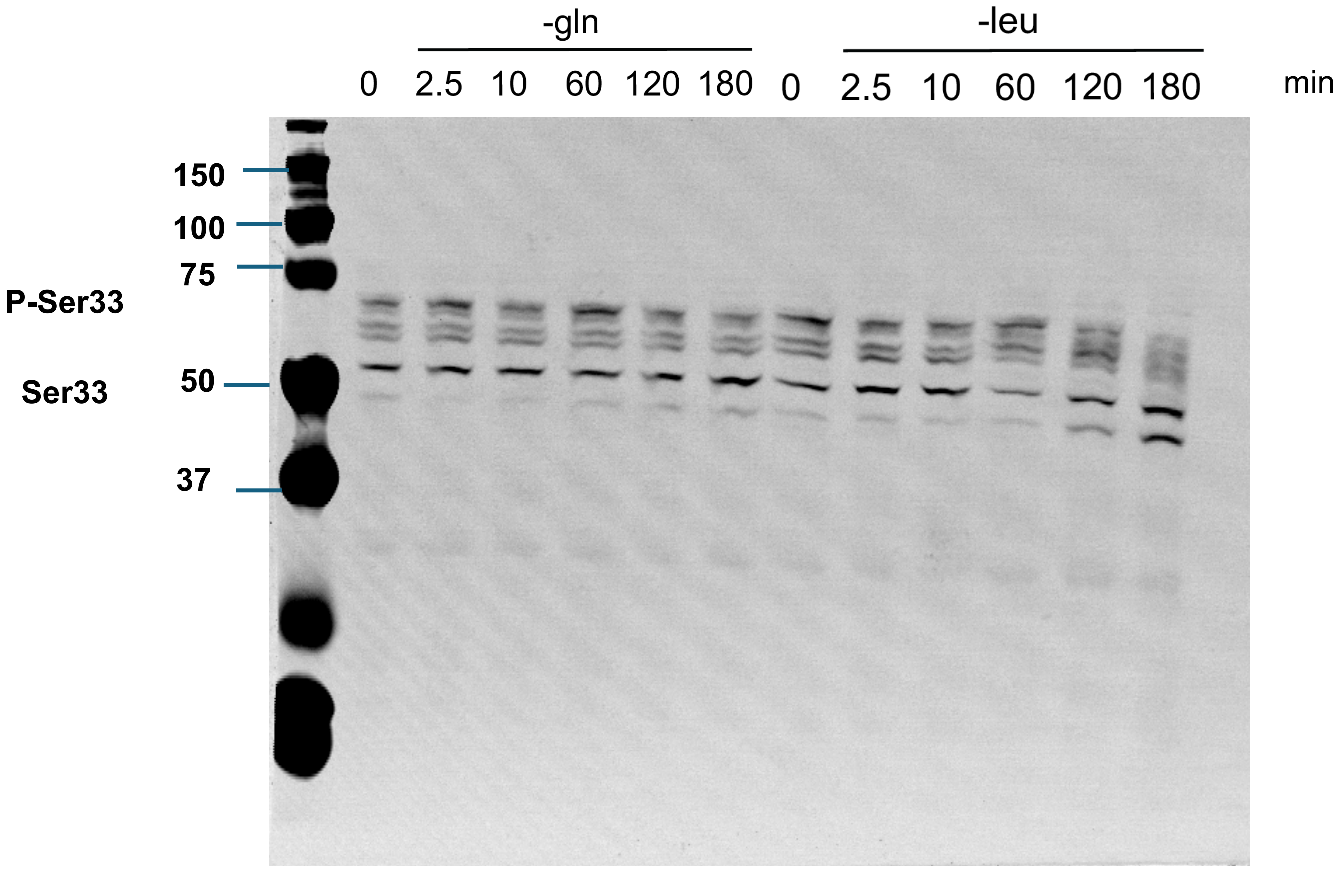

-gln
-leu
0
2.5
10
60
120
180
0
2.5
10
60
120
180
min
150
100
75
P-Ser33
50
Ser33
37

Figure 5F

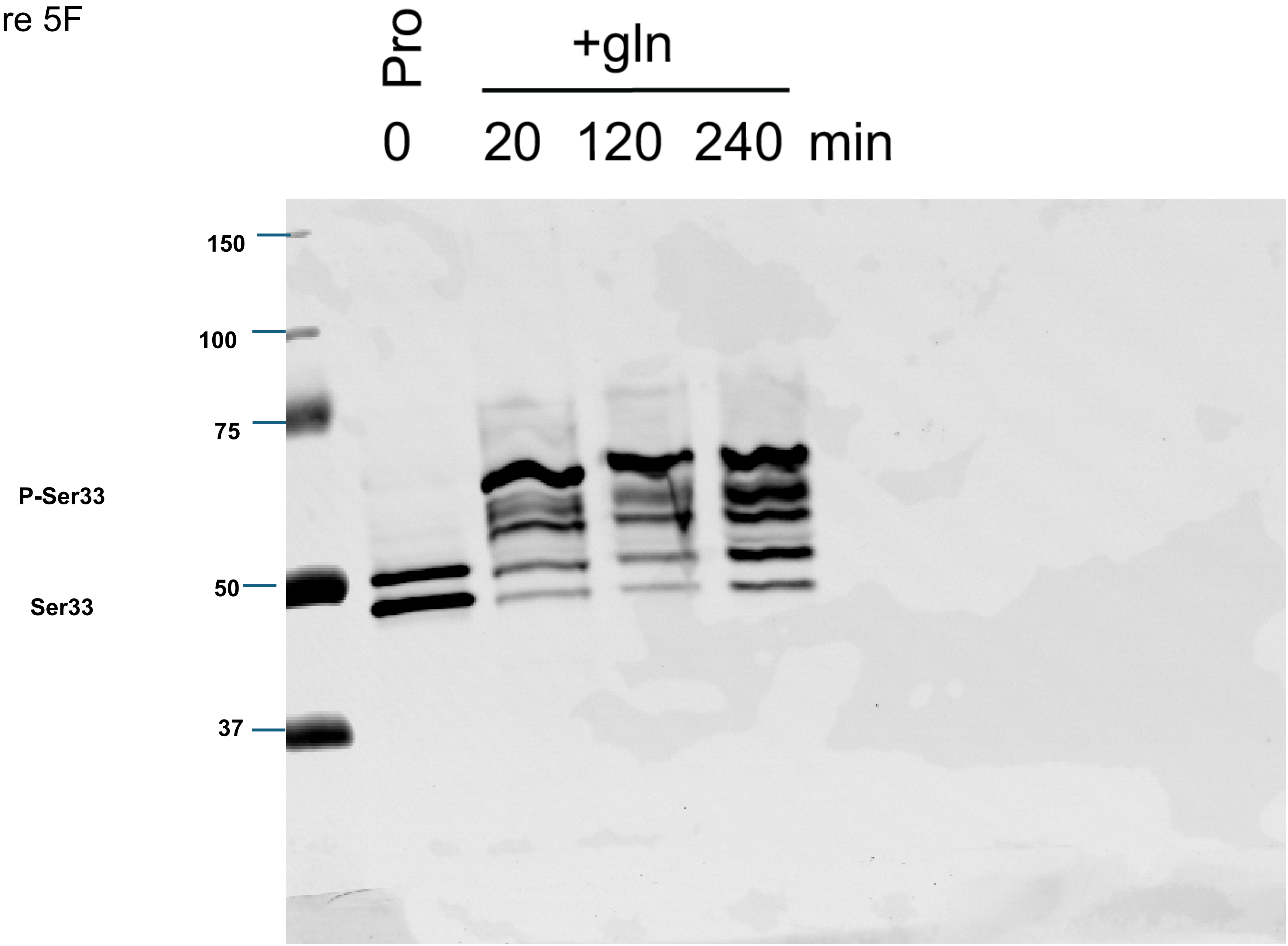

Figure 5G

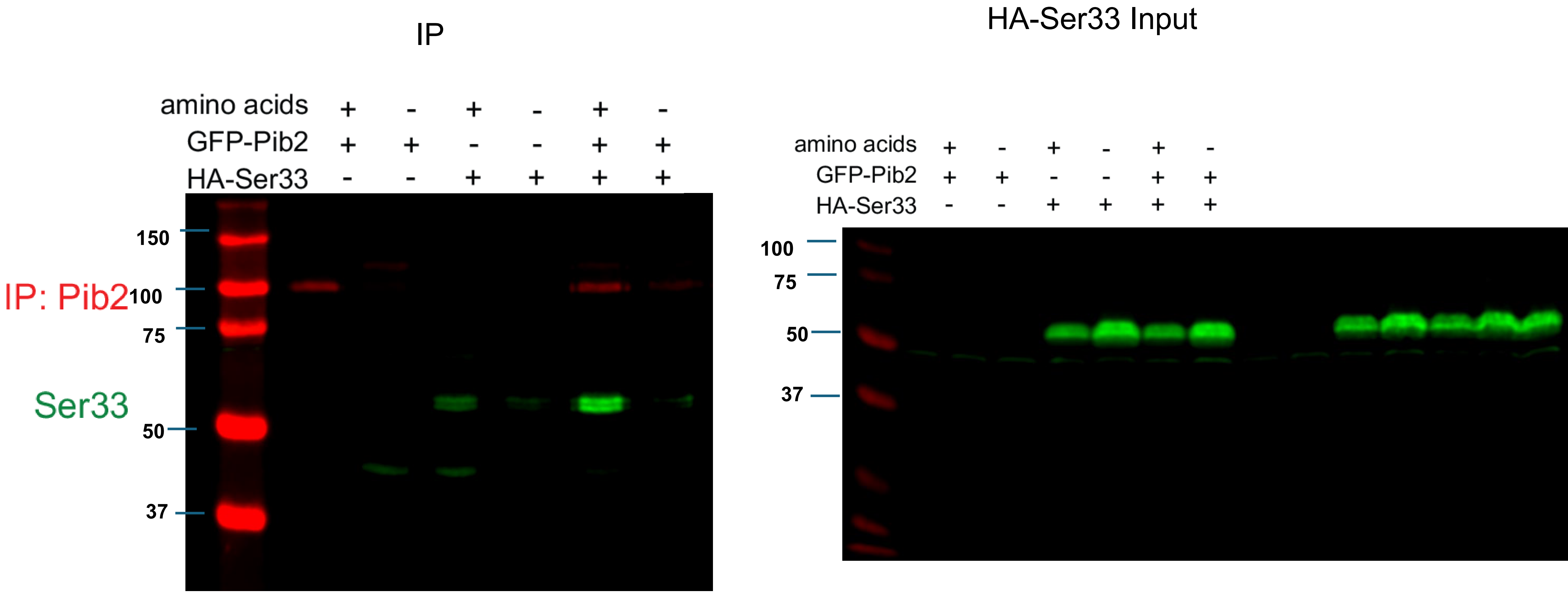
IP
amino acids + - + - + -
GFP-Pib2 + + - - + +
HA-Ser33 - - + + + +
150
IP: Pib2
100
75
Ser33
50
37
HA-Ser33 Input
amino acids + - + - + -
GFP-Pib2 + + - - + +
HA-Ser33 - - + + + +
100
75
50
37

Figure 5G cont

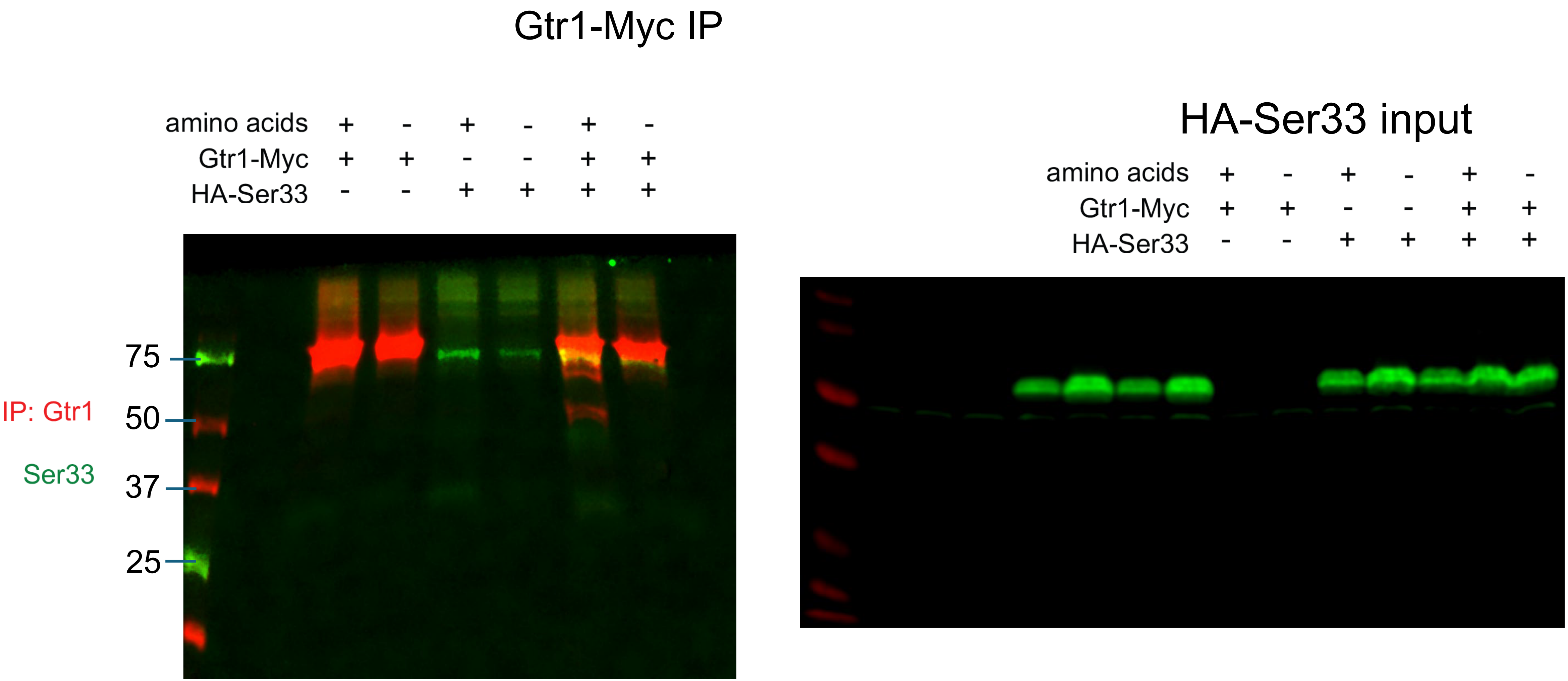

Supplement: Figure 5—source data 1. [file elife-94628-fig5-data1.pdf]
